# Cold-induced hepatocyte-derived exosomes activate brown adipose thermogenesis via miR-293-5p-mediated transcriptional reprogramming

**DOI:** 10.1038/s41420-025-02697-1

**Published:** 2025-08-22

**Authors:** Xiujuan Gao, Junqing Xu, Zengqiang Xu, Mengxin Jiang, Jiahao Zhu, Yang Geng, Shengjun Dong, Yanuo Li, Zhengtong Zhou, Yingjiang Xu

**Affiliations:** 1 Binzhou Medical University Hospital, Binzhou City, PR China; 2 The First School of Clinical Medicine, Binzhou Medical University, Binzhou City, PR China; 3 Department of Pathophysiology, School of Basic Medicine, Binzhou Medical University, Yantai City, PR China; 4 Institute of Medical Genomics, Biomedical Sciences College & Shandong Medicinal Biotechnology Centre, Medical Science and Technology Innovation Center, Shandong First Medical University & Shandong Academy of Medical Sciences, Jinan City, PR China

**Keywords:** Gene expression, DNA metabolism

## Abstract

The liver-adipose axis represents a crucial regulatory network that governs systemic lipid homeostasis, with signals originating from the liver orchestrating the plasticity of adipose tissue through diverse mechanisms. A comprehensive understanding of these bidirectional communication pathways may uncover novel therapeutic approaches for metabolic disorders. Our research demonstrates that exposure to cold stimulates the liver to secrete exosomes, which enhance thermogenic activation in adipose tissue, as observed in both in vitro and in vivo models. This enhancement of thermogenesis is mechanistically associated with the cold-induced upregulation of hepatocyte-derived exosomal miR-293-5p. Importantly, the pharmacological administration of a miR-293-5p agomir significantly mitigates diet-induced obesity and related metabolic dysfunctions in murine models. Through mechanistic analysis, we identified Tet1 as a direct downstream target of miR-293-5p, noting that the ectopic expression of Tet1 disrupts the thermogenic programming of brown adipose tissue (BAT) independently of miR-293-5p modulation. Our findings establish cold-activated hepatocyte exosomes as endocrine signaling mediators that carry thermogenic microRNA cargos, with miR-293-5p emerging as a key regulator.

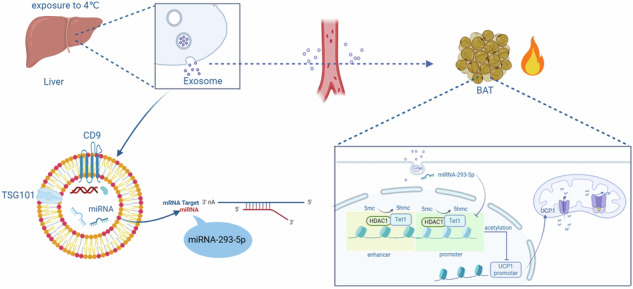

## Introduction

Adipose tissue, recognized as a dynamic endocrine organ, plays a crucial role in maintaining systemic energy homeostasis through its two functional components. Brown adipose tissue (BAT) is characterized by unique morphological attributes, including dense vascular networks, extensive sympathetic innervation, and multilocular adipocytes with cytoplasm rich in mitochondria. The thermogenic capacity of BAT is primarily facilitated by uncoupling protein 1 (UCP1)-mediated non-shivering thermogenesis, which dissipates chemical energy as heat via mitochondrial proton leakage [1–3]. Conversely, white adipose tissue (WAT) exhibits significant plasticity through its ability to undergo “browning,” a process induced by cold exposure, exercise, or specific agonists, resulting in the formation of UCP1-positive beige adipocytes. These cells express canonical BAT markers such as *Cidea, Cox7a, Prdm16*, and *Pgc-1α*, thereby acquiring thermogenic capabilities [4, 5]. This adaptive thermogenic reprogramming, characterized by metabolic flexibility and inducibility, holds substantial therapeutic promise for addressing metabolic disorders.

The autonomic nervous system functions as the primary regulator of BAT activation, wherein cold-induced sympathetic stimulation prompts the release of norepinephrine, subsequently initiating β3-adrenergic receptor signaling [6]. In addition to traditional adrenergic pathways, recent evidence underscores a sophisticated regulatory network comprising paracrine and endocrine factors, as well as metabolite signaling, which facilitates inter-tissue communication [7]. Although significant research has elucidated the transcriptional cascades following β3-AR activation, the spatiotemporal integration of non-canonical signaling pathways remains inadequately characterized.

Extracellular vesicles (EVs), particularly exosomes, have emerged as pivotal mediators of interorgan communication in the regulation of metabolism. These nanoscale carriers facilitate the transfer of microRNAs (miRNAs) between tissues, with their cargo profiles serving as indicators of physiological status. Under obesogenic conditions, there is a significant alteration in the miRNA signatures of plasma exosomes, exemplified by the upregulation of miR-122, miR-192, and miR-27a-3p/b-3p, which can propagate metabolic dysfunction to recipient tissues [8]. Studies utilizing adipocyte-specific Dicer knockout models (ADicerKD) and observations from human lipodystrophy cases underscore the essential role of adipose-derived exosomal miRNAs in physiological tissue remodeling [9]. Notably, endogenous adipocyte miRNAs such as miR-34a-5p and miR-155 play differential roles in the regulation of brown/beige adipogenesis by targeting Fgfr1/Klb/Sirt1 and modulating the transitions between proliferation and differentiation, respectively [10, 11]. The possibility of extra-adipose origins for these regulatory miRNAs via exosomal trafficking merits further investigation.

As the central metabolic hub, the liver intricately regulates nutrient partitioning through the dynamic coordination of glucose and lipid fluxes [12, 13]. This study elucidates a previously unrecognized communication axis between the liver and BAT, mediated by hepatocyte-derived exosomes (hepExos). Exposure to cold conditions stimulates the hepatic secretion of exosomes enriched with miR-293-5p, which enhances the thermogenic capacity of BAT both in vitro and in vivo. Through integrated miRNA sequencing and computational analyses, Ten-eleven translocation methylcytosine dioxygenase 1 (Tet1) was identified as a direct target of miR-293-5p. This discovery of the hepExo-miRNA-Tet1 regulatory axis provides novel mechanistic insights into interorgan metabolic communication and identifies potential therapeutic targets for metabolic syndrome.

## Results

### Exosomes from cold-activated hepatocytes enhance BAT thermogenesis

To examine the thermogenic regulatory potential of hepatic exosomes, 10-week-old C57BL/6 mice maintained on a standard diet were subjected to either 4 °C or room temperature (RT) for a duration of three days. Primary hepatocytes were subsequently isolated from the mice exposed to cold conditions, and hepExos were purified using an optimized protocol (Supplementary Fig. 1A). Quantitative analysis indicated a significantly higher yield of exosomes from cold-exposed hepatocytes compared to those maintained at RT (Supplementary Fig. 1B). Transmission electron microscopy (TEM) confirmed the characteristic cup-shaped morphology of the isolated vesicles (Supplementary Fig. 1C), and western blot analysis verified the enrichment of exosomal markers CD9 and TSG101 (Supplementary Fig. 1D), collectively affirming the successful isolation of hepExos.

Ten-week-old male C57BL/6 mice, maintained on a standard chow diet, were subjected to a 72 h cold acclimation at 4 °C or maintained at room temperature (25 °C) as a control. Primary hepatocytes were isolated through collagenase perfusion digestion and subsequently cultured under serum-free conditions. HepExos were extracted from the conditioned media via sequential ultracentrifugation and sucrose density gradient purification (Supplementary Fig. 1A). Quantitative analysis indicated a 1.8-fold increase in exosome yield from hepatocytes exposed to cold conditions compared to the control group (Supplementary Fig. 1B). Transmission electron microscopy confirmed the presence of vesicles measuring 80-150 nm, exhibiting cup-shaped morphology and double-membrane structures (Supplementary Fig. 1C). Immunoblotting analysis demonstrated a significant enrichment of canonical exosomal markers, such as CD9 and TSG101, with minimal contamination from proteins associated with cellular organelles (Supplementary Fig. 1D). This protocol underscores the methodological robustness for isolating intact HepExos while preserving their structural integrity and molecular characteristics.

**Fig. 1:**
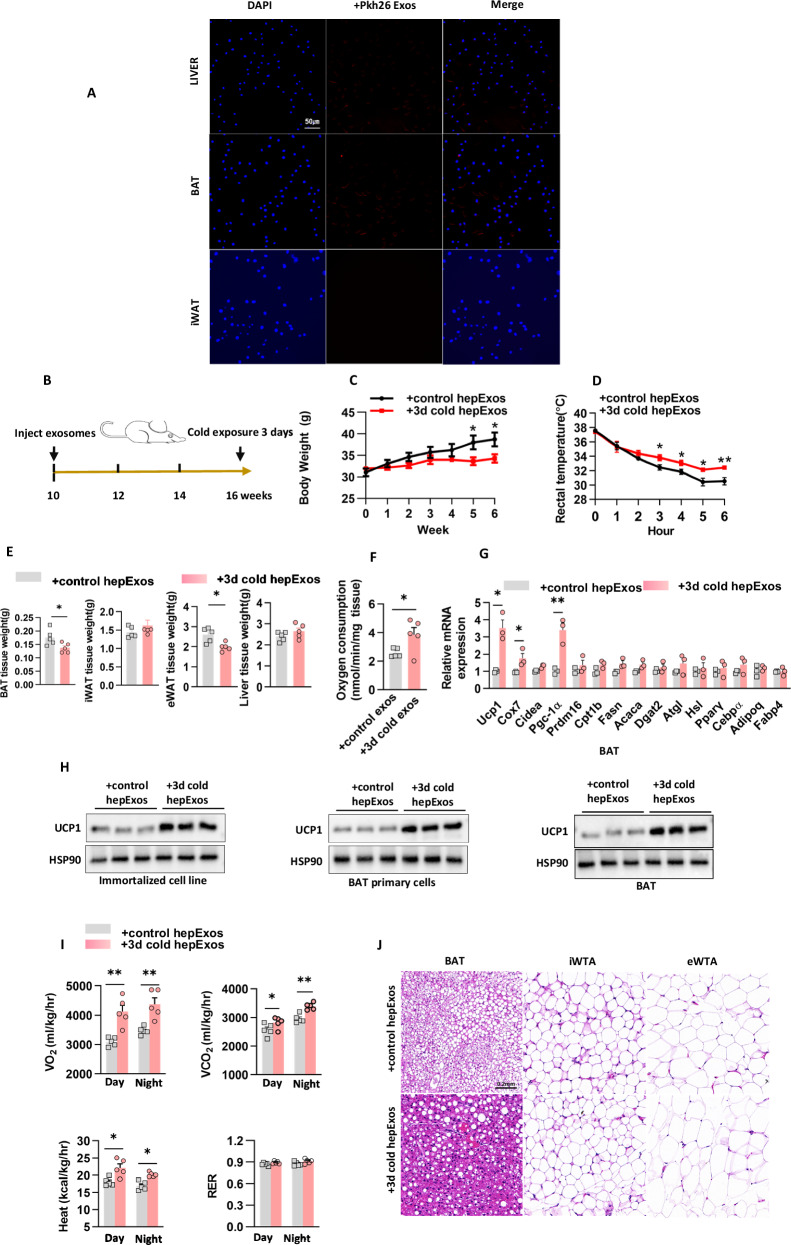
Exosomes from cold-activated hepatocytes enhance BAT thermogenesis. **A** HepExos were successfully delivered to the target BAT. Scale bar: 200 μm. **B** The in vivo experimental protocol involved the administration of cold-exposed exosomes via tail vein injection. **C** Body weight measurements of the mice in group (**B**) were recorded, with n = 5 mice per group. **D** Rectal temperature was assessed in mice from group (**B**) when exposed to a temperature of 4°C, with n = 5 mice per group. **E** The mass of fat and liver tissues was measured in mice from group (**B**), with n = 5 mice per group. **F** The oxygen consumption rate in BAT was determined using Clark electrodes, as described for group (**B**), across n = 5 independent experiments. **G** Gene expression in BAT was evaluated through quantitative PCR, as conducted in group (**B**), with n = 5 independent experiments. **H** UCP1 protein levels were quantified in immortalized brown adipocytes, primary BAT cells, and BAT tissue, with n = 3 independent experiments. **I** C57BL/6 J male mice underwent indirect calorimetry analysis under basal conditions, with n = 5 mice per group. **J** Hematoxylin and eosin staining was performed on BAT samples from mice in group (**B**). Scale bar: 0.2 mm for BAT, iWAT, and eWAT.

Exosomes, functioning as nanoscale mediators of intercellular communication, facilitate metabolic coordination by transferring bioactive cargo such as miRNAs, proteins, and lipids [14, 15]. To examine the biodynamics of hepExos, PKH26-labeled particles (1 × 10^9^/mouse) were administered intravenously to 10-week-old C57BL/6 J wild-type (WT) mice. Fluorescence microscopy conducted 16 h post-injection revealed selective biodistribution, with a pronounced tropism for hepatic tissue and BAT (Fig. 1A). Importantly, the inguinal white adipose tissue (iWAT) displayed undetectable signal intensity, indicating tissue-specific exosomal trafficking. These findings conclusively identify BAT as a preferential recipient of circulating hepExos.

To explore the potential role of hepExos in the regulation of thermogenesis in BAT, we administered hepExos intravenously to 10-week-old WT mice at a dosage of 5×10^9^ particles per mouse, twice weekly for a duration of six weeks. Exosomes derived from mice adapted to room temperature were used as controls (Fig. 1B). Notably, mice treated with hepExos from cold-adapted mice (exposed to 4 °C for 3 days) exhibited reduced body weight gain (Fig. 1C) and improved cold tolerance (Fig. 1D), despite similar food and water intake and serum triglyceride levels between the experimental and control groups (Supplementary Fig. 2A–C). These metabolic enhancements were associated with significant decreases in BAT mass and epididymal white adipose tissue (eWAT) weight (Fig. 1E), as well as increased oxygen consumption rates in BAT (Fig. 1F). Molecular analyses indicated that hepExos from cold-adapted mice specifically upregulated the expression of thermogenic genes in BAT, including *Ucp1, Cox7a*, and *Pgc-1α*, without affecting iWAT (Fig. 1G, Supplementary Fig. 2D). Consistently, the levels of UCP1 protein were significantly elevated in BAT (Fig. 1H), while remaining unchanged in iWAT (Supplementary Fig. 2E). Whole-animal metabolic profiling using indirect calorimetry demonstrated that treatment with cold-adapted hepExos increased basal heat production and oxygen consumption.

In addition to these in vivo observations, we conducted a systematic evaluation of the cell-autonomous effects of cold-adapted hepExos in vitro. Utilizing a standardized protocol, both immortalized brown adipocytes and primary brown adipocytes were exposed to cold-adapted hepExos (1 × 10^8^ particles per 0.5 × 10^6^ cells) in an exosome-free differentiation medium for a duration of 48 hours. Notably, quantitative PCR analysis indicated an upregulation of core thermogenic effectors across all adipocyte models (Fig. 1H). These findings collectively suggest that hepExos derived from cold-exposed mice enhance the thermogenic program in BAT.

### Hepatocyte exosomal miRNAs mediate metabolic communication between organs

Building upon established evidence regarding the pivotal role of YBX1 in the packaging of exosomal miRNA [16, 17], we utilized liver-specific *Ybx1* knockdown to investigate miRNA functionality within hepExos. C57BL/6 mice were administered tail vein injections of AAV9-shYbx1 or control AAV9-Ctrl (2 × 10^11^ vector genomes in 200 μL PBS, with booster injections administered every four weeks). After a period of 10 weeks on a standard chow diet and a subsequent 3-day cold acclimation at 4 °C (Supplementary Fig. 3A), quantitative RT-PCR analysis revealed an approximate 70% reduction in hepatic Ybx1 mRNA levels (Supplementary Fig. 3B). Importantly, the knockdown of *Ybx1* did not impact the production of hepExos (Supplementary Fig. 3C). However, miRNA quantification indicated an 80% depletion in hepExos, as demonstrated by the levels of miR-122, a hepatocyte-enriched miRNA that serves as a cargo biomarker (Supplementary Fig. 3D).

Subsequent assessments of cold tolerance in AAV9-YBX1 knockdown (KD) mice revealed significantly lower core body temperatures compared to AAV9-Ctrl KD mice (Supplementary Fig. 3E). Deficiency in *Ybx1* led to an increase in body weight (Supplementary Fig. 3F) and elevated serum triglyceride levels (Supplementary Fig. 3G). Importantly, oxygen consumption rates in BAT were reduced in KD mice (Supplementary Fig. 3H). Transcriptional profiling indicated impaired thermogenesis in BAT, with expressions of *Ucp1, Cidea, Pgc-1α*, and *Prdm16* reduced by 30-52% (Supplementary Fig. 3I). This molecular suppression was associated with a decrease in UCP1 protein abundance (Supplementary Fig. 3J). Comprehensive metabolic analyses confirmed systemic impairments, including reductions in heat production, VO_2_, and VCO_2_ (Supplementary Fig. 3K). Histological examination showed that BAT from KD mice exhibited lipid droplets that were twice as large, whereas the morphology of white adipose tissue remained similar between groups (Supplementary Fig. 3L). Collectively, these findings establish YBX1 as a critical regulator of hepatic exosome-mediated thermogenic programming in BAT during cold adaptation.

**Fig. 2:**
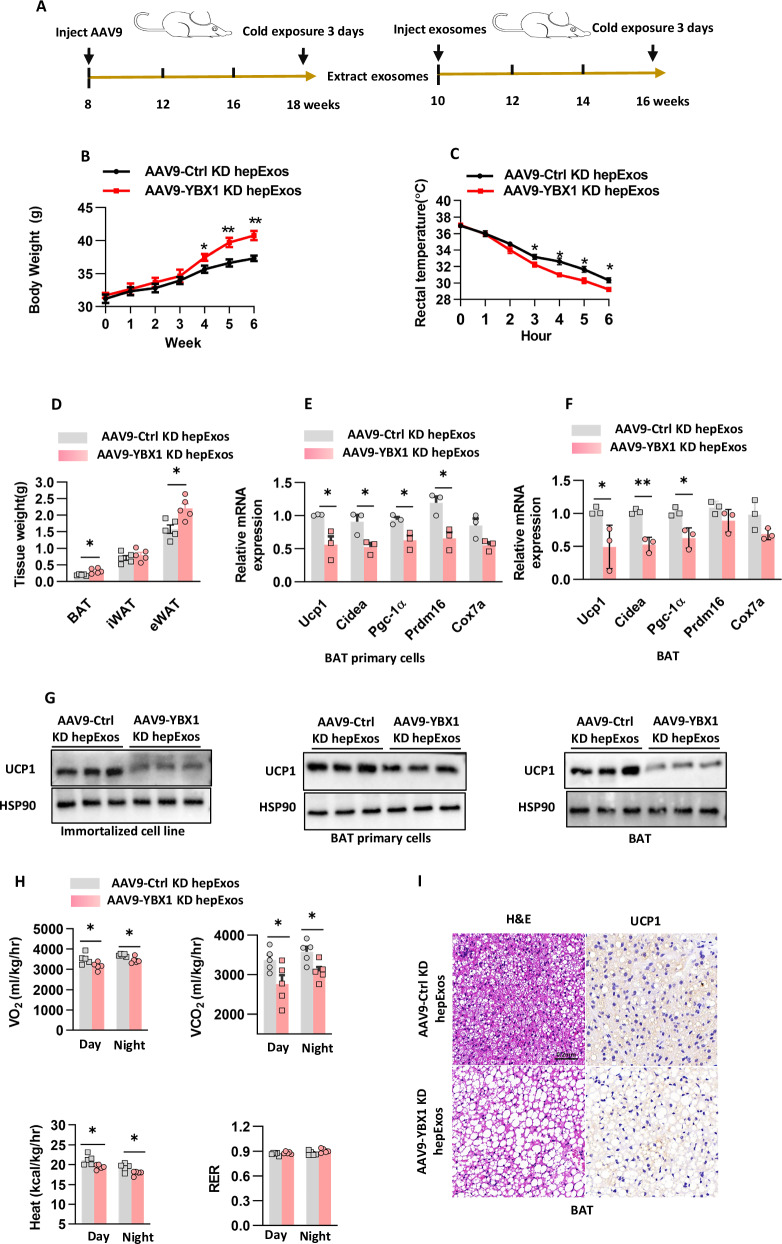
Hepatocyte exosomal miRNAs mediate metabolic communication between organs. **A** The in vivo protocol involved the intravenous injection of AAV9-Ctrl KD hepExos and AAV9-YBX1 KD hepExos. The following parameters were assessed: **B** The body weight of the mice from protocol (**A**), with n = 5 mice per group. **C** Rectal temperature measurements were taken when the mice from protocol (**A**) were exposed to a 4 °C environment, with n = 5 mice per group. **D** The fat mass weight of the mice from protocol (**A**), with n = 5 mice per group. **E** Gene expression in BAT primary cells was analyzed using quantitative PCR, as described in protocol (**A**), with n = 3 independent experiments. **F** Gene expression in BAT was analyzed using quantitative PCR, as described in protocol (**A**), with n = 5 independent experiments. **G** UCP1 protein levels were analyzed in immortalized brown adipocytes, BAT primary cells, and BAT, with n = 3 independent experiments. **H** C57BL/6 J male mice underwent indirect calorimetry analysis in the basal state, with n = 5 mice per group. **I** Hematoxylin and eosin (H&E) staining and UCP1 protein expression were detected by immunohistochemistry in the mice from protocol (**A**) across different treatment groups. The scale bar for BAT is 0.2 mm.

In alignment with the effects of endogenous *Ybx1* deficiency, administration of AAV9-YBX1 KD hepExos significantly disrupted the thermogenic programming of BAT. Within our experimental framework (Fig. 2A), WT mice receiving biweekly intravenous injections of 5 × 10^9^ KD hepExos over a six-week period exhibited similar ad libitum food intake and serum triglyceride levels compared to control mice (Supplementary Fig. 4A-C). Notably, mice treated with YBX1 KD hepExos demonstrated accelerated weight gain and reduced cold tolerance (Fig. 2B, C). This metabolic disturbance was associated with increased ectopic fat accumulation, particularly in the eWAT mass (Fig. 2D). Molecular analysis indicated a systemic downregulation of thermogenic machinery, as evidenced by decreased mRNA levels of *Ucp1, Pgc-1α*, and *Cidea* in BAT (Fig. 2E, F), alongside reduced UCP1 protein expression (Fig. 2G). Importantly, these effects were specific to BAT, as the morphology and gene expression of inguinal white adipose tissue (iWAT) remained unchanged (Supplementary Fig. 4D, E). Indirect calorimetry further quantified the metabolic impairment, revealing decreased oxygen consumption (VO_2_) and reduced heat production (Fig. 2H). Histological examination of BAT from KD hepExos-treated mice showed hypertrophic lipid droplets and a reduced number of UCP1-positive multilocular adipocytes (Fig. 2I). These findings demonstrate that hepExos function as crucial signaling mediators in the thermogenic response to cold exposure in mice.

### miR-293-5p plays a crucial role in regulating lipid homeostasis within adipocytes

**Fig. 3:**
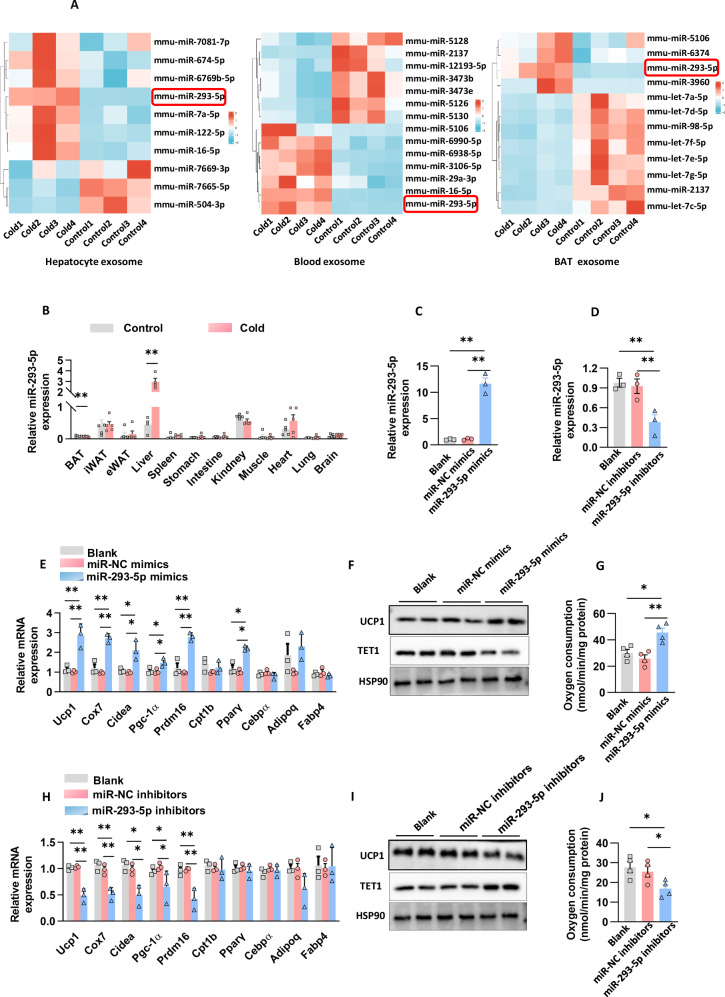
miR-293-5p plays a crucial role in regulating lipid homeostasis within adipocytes. **A** The differential expression of miRNAs was assessed in exosomes derived from hepatocytes, blood, and BAT of mice exposed to cold temperatures. **B** The fold change in miR-293-5p expression levels was evaluated across multiple tissues following cold exposure, with a sample size of n = 5 mice per group. **C** Immortalized brown adipocytes were differentiated into mature adipocytes with the addition of a control, miR-NC mimics, and miR-293-5p mimics on the third day of differentiation. The fold change in miR-293-5p expression levels was analyzed on the sixth day of differentiation, based on n = 3 independent experiments. **D** Immortalized brown adipocytes were differentiated into mature adipocytes with the addition of a control, miR-NC inhibitors, and miR-293-5p inhibitors on the third day of differentiation. The fold change in miR-293-5p expression levels was analyzed on the sixth day of differentiation, with n = 3 independent experiments. **E** Gene expression analysis was conducted on the sixth day of differentiation as described in section C, with n = 3 independent experiments. **F** Protein levels of UCP1 and TET1 were analyzed on the sixth day of differentiation as described in section C, with n = 2 independent experiments. **G** The oxygen consumption rate was measured using Clark electrodes as described in section C, with n = 3 independent experiments. **H** Gene expression analysis was conducted on the sixth day of differentiation as described in section D, with n = 3 independent experiments. **I** The levels of UCP1 and TET1 proteins were assessed on day 6, following the methodology outlined in (**D**), with data derived from two independent experiments. **J** The oxygen consumption rate was determined using Clark electrodes, as described in (**D**), with data obtained from three independent experiments.

To elucidate the bioactive mediators involved in hepatic exosome-mediated lipid regulation, we concentrated on miRNA cargoes due to their emerging roles in orchestrating adipose metabolism and inflammatory signaling in BAT. Through small RNA sequencing of hepExos, miR-293-5p was identified as the most significantly upregulated miRNA in the liver, circulation, and BAT-derived exosomes following cold exposure (Fig. 3A, Table S1). Notably, this miRNA was the only candidate exhibiting concurrent elevation across all three compartments. Quantitative RT-PCR profiling revealed that cold exposure specifically induced upregulation of miR-293-5p in hepatic tissue exosomes, with no significant changes observed in BAT (Fig. 3B). This compartmentalized expression pattern strongly supports the existence of a hepatic exosomal miRNA-adipose axis that mediates interorgan metabolic communication. The spatiotemporal dynamics and tissue specificity of miR-293-5p elevation prompted a focused investigation into its metabolic regulatory functions.

To elucidate the metabolic regulatory function of miR-293-5p, we conducted gain- and loss-of-function experiments during in vitro adipocyte differentiation. Transfection with miR-293-5p mimics resulted in substantial overexpression, whereas the use of inhibitors significantly decreased endogenous levels, as determined by RT-qPCR (Fig. 3C-D). The transfection with mimics specifically led to an upregulation of thermogenic gene expression in immortalized brown adipocytes, with significant induction observed in *Ucp1, Cox7a, Cidea, Pgc-1α*, and *Prdm16*, while lipogenic markers (*Cebpα, Adipoq, Fabp4*) and the β-oxidation gene *Cpt1b* remained unchanged (Fig. 3E). Although there was a moderate upregulation of *Pparγ*, early-stage clonal expansion was unaffected (Supplementary Fig. 5A). The enhancement of thermogenesis was further supported by an increase in UCP1 protein levels (Fig. 3F) and an elevated mitochondrial oxygen consumption rate (Fig. 3G). In contrast, treatment with inhibitors maintained adipogenic differentiation markers (Fig. 3H) and proliferative capacity (Supplementary Fig. 5B) but significantly impaired thermogenic capacity, as evidenced by reduced UCP1 protein levels (Fig. 3I) and suppressed expression of thermogenic transcripts (*Ucp1, Cox7a, Cidea, Pgc-1α, Prdm16*) (Fig. 3H), ultimately leading to a decrease in OCR (Fig. 3J). These complementary findings identify miR-293-5p as a pivotal and specific regulator of thermogenic programming in adipocytes, functioning independently of the broader adipogenic differentiation processes.

### Exosomal miR-293-5p from primary hepatocytes triggers the thermogenic program in adipocytes

**Fig. 4:**
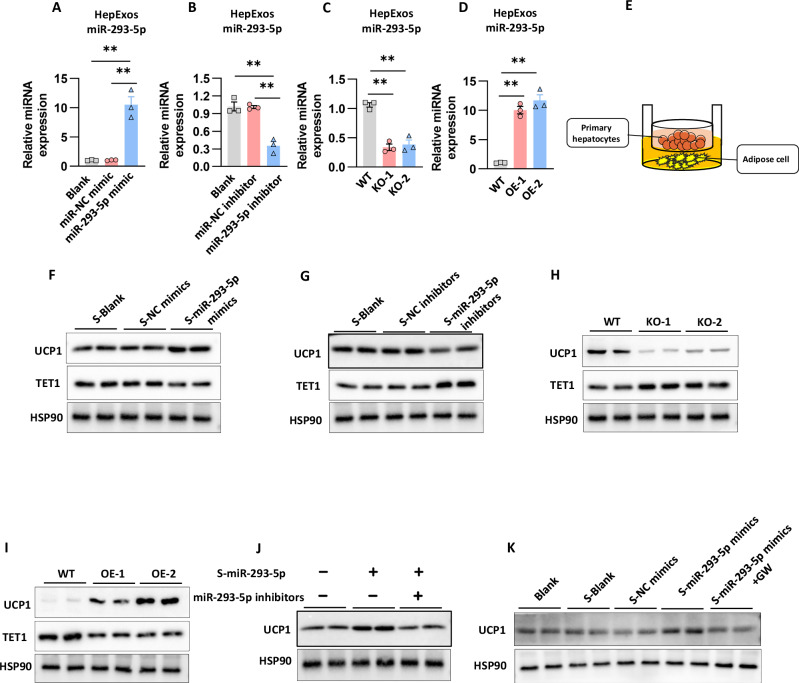
Exosomal miR-293-5p from primary hepatocytes triggers the thermogenic program in adipocytes. The study investigates miR-293-5p levels in exosomes derived from the culture medium of primary hepatocytes under various conditions. Specifically, the experimental conditions include: **A** Hepatocytes transfected with either a miR-NC mimic or a miR-293-5p mimic, with data collected from three independent experiments. **B** Hepatocytes transfected with either a miR-NC inhibitor or a miR-293-5p inhibitor, also based on three independent experiments. **C** Hepatocytes infected with lentiviral shRNAs targeting miR-293-5p, with three independent experiments conducted. **D** Hepatocytes infected with lentiviruses overexpressing miR-293-5p, with data from three independent experiments. **E** A schematic representation of the transwell coculture chamber is provided. **F**, **G** Primary hepatocytes were transfected with either miR-293-5p mimics, inhibitors, or controls. The resulting culture medium was harvested for further analysis. Western blot analyses were performed to assess UCP1 and TET1 expression in immortalized brown adipocytes incubated with the conditioned medium, with results from two independent experiments. **H**, **I** Primary hepatocytes were subjected to infection with lentiviral vectors carrying shRNAs targeting miR-293-5p or with lentiviral vectors overexpressing miR-293-5p. Subsequently, the specified culture medium was collected for further analysis. Western blot assays were conducted to assess the expression levels of UCP1 and TET1 in immortalized brown adipocytes incubated with the conditioned medium. The experiments were performed independently twice n = 2. **J** Western blot analysis was performed to evaluate UCP1 expression in immortalized brown adipocytes that were transfected with a miR-293-5p inhibitor for 24 h, followed by incubation with conditioned medium derived from primary hepatocytes overexpressing miR-293-5p for 48 h. The experiments were conducted independently twice n = 2. **K** Western blot analysis was carried out to determine UCP1 expression in immortalized brown adipocytes incubated with conditioned medium from the specified primary hepatocytes. The experiments were performed independently twice n = 2.

The secretion of exosomes in adipocytes is a highly regulated process that involves multiple steps governed by specific molecular machinery [18]. To investigate the regulatory role of miR-293-5p in the biology of hepExos, we developed a controlled secretion model using primary hepatocytes. Transfection with miR-293-5p mimics resulted in an approximately 10-fold increase in exosomal miR-293-5p levels compared to scramble controls (Fig. 4A), while the use of inhibitors led to a 30% reduction in exosomal content (Fig. 4B). Lentiviral-mediated knockout (KO-1/KO-2) and overexpression (OE-1/OE-2) systems exhibited dose-dependent modulation of exosomal miR-293-5p levels (Fig. 4C, D), as confirmed by nanoparticle tracking analysis and quantitative reverse transcription PCR (qRT-PCR).

In a transwell coculture system designed to model hepatic-adipose interaction (Fig. 4E), hepatocyte-derived exosomes enriched with miR-293-5p, following mimic transfection, resulted in the upregulation of UCP1 in brown adipocytes (Fig. 4F). In contrast, the treatment of hepatocytes with an inhibitor led to a reduction in adipocyte UCP1 expression (Fig. 4G). This suppression was further corroborated by genetic manipulation using lentiviral knockout techniques (Fig. 4C), which decreased UCP1 expression in control groups (Fig. 4H). Conversely, overexpression systems (OE-1/OE-2) were observed to enhance UCP1 levels (Fig. 4I). These findings, derived from both pharmacological and genetic methodologies, collectively establish hepatocyte-derived exosomal miR-293-5p as a significant regulator of adipocyte thermogenesis, functioning through exosome-mediated intercellular communication.

To further substantiate the role of hepExos-miR-293-5p in thermogenic regulation, we undertook a series of functional assays. Adipocytes were initially pre-incubated with a specific miR-293-5p inhibitor before being exposed to conditioned media from miR-293-5p-overexpressing primary hepatocytes. Notably, this pharmacological intervention completely negated the upregulation of UCP1 expression elicited by hepatocyte-derived factors (Fig. 4J), thereby underscoring the miRNA’s pivotal role in mediating this regulatory effect. To specifically investigate the involvement of EVs, we utilized GW4869, a well-established inhibitor of exosome biogenesis and secretion. The pharmacological inhibition of EV production significantly attenuated the thermogenic activation in adipocytes treated with media from miR-293-5p-overexpressing hepatocytes (Fig. 4K). These complementary experimental strategies provide compelling evidence that hepExos function as critical vectors for miR-293-5p delivery, and that this intercellular communication mechanism is essential for initiating thermogenic programming in adipocytes.

### Agomir miR-293-5p injection triggers thermogenesis in BAT

**Fig. 5:**
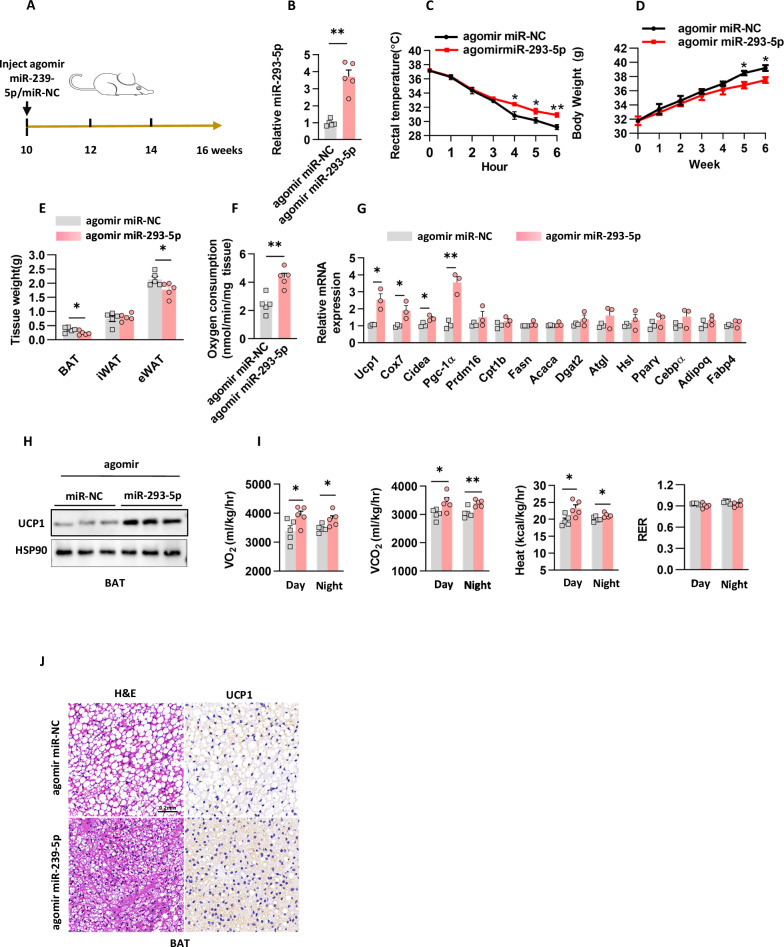
Agomir miR-293-5p injection triggers thermogenesis in BAT. **A** The in vivo protocol involved the administration of agomir miR-293-5p or miR-NC via tail vein injection. **B** The levels of miR-293-5p in the BAT of mice from protocol (**A**) were quantified, with n = 5 mice per group. **C**. Rectal temperature was recorded when the mice from protocol (**A**) were exposed to a temperature of 4°C, with n = 5 mice per group. **D** The body weight of mice subjected to protocol (**A**) was measured, with n = 5 mice per group. **E** The fat mass weight of mice from protocol (**A**) was assessed, with n = 5 mice per group. **F** The oxygen consumption rate in BAT was determined using Clark electrodes, as described in protocol (**A**), with n = 5 independent experiments. **G** Gene expression in BAT was analyzed via quantitative PCR, following protocol (**A**), with n = 3 independent experiments. **H**. UCP1 protein levels in BAT were evaluated, with n = 3 independent experiments. **I** Male C57BL/6 J mice underwent indirect calorimetry analysis under basal conditions, with n = 5 mice per group. **J** Hematoxylin and eosin (H&E) staining and UCP1 protein expression were assessed through immunohistochemistry in BAT from mice subjected to different treatment groups in protocol (**A**). The scale bar for BAT is 0.2 mm.

To examine the in vivo role of miR-293-5p in thermogenic regulation, wild-type mice were administered weekly intravenous injections of agomir miR-293-5p or a control miR-NC over a period of six weeks (Fig. 5A). Pharmacological activation resulted in a threefold increase in miR-293-5p levels in BAT compared to controls (Fig. 5B), without affecting baseline metabolic parameters such as food and water intake, body weight progression, or serum lipid profiles (Supplementary Fig. 6A–C). Importantly, the enhancement of miR-293-5p led to progressive metabolic adaptations, which were characterized by improved cold tolerance, delayed onset of body weight gain primarily due to a reduction in eWAT mass, and an increase in OCR in BAT (Fig. 5C–F).

Molecular analyses have demonstrated a coordinated upregulation of thermogenic effectors in BAT, characterized by an increase in UCP1 protein levels and the transcriptional activation of mitochondrial uncoupling genes, including *Ucp1, Cox7a, Cidea*, and *Pgc-1α* (Fig. 5G and 5H). Whole-body calorimetry indicated an increase in energy expenditure, which was accompanied by morphological remodeling of BAT, evidenced by a reduction in lipid droplet size and a dense accumulation of UCP1-positive adipocytes (Fig. 5I and 5J). The tissue-specific nature of this response is highlighted by the minimal changes in gene expression observed in iWAT. Collectively, these multimodal data identify miR-293-5p as a critical enhancer of BAT-driven thermogenesis, mediated through mitochondrial activation and adipose tissue remodeling.

### miR-293-5p therapy slowed obesity in mice

**Fig. 6:**
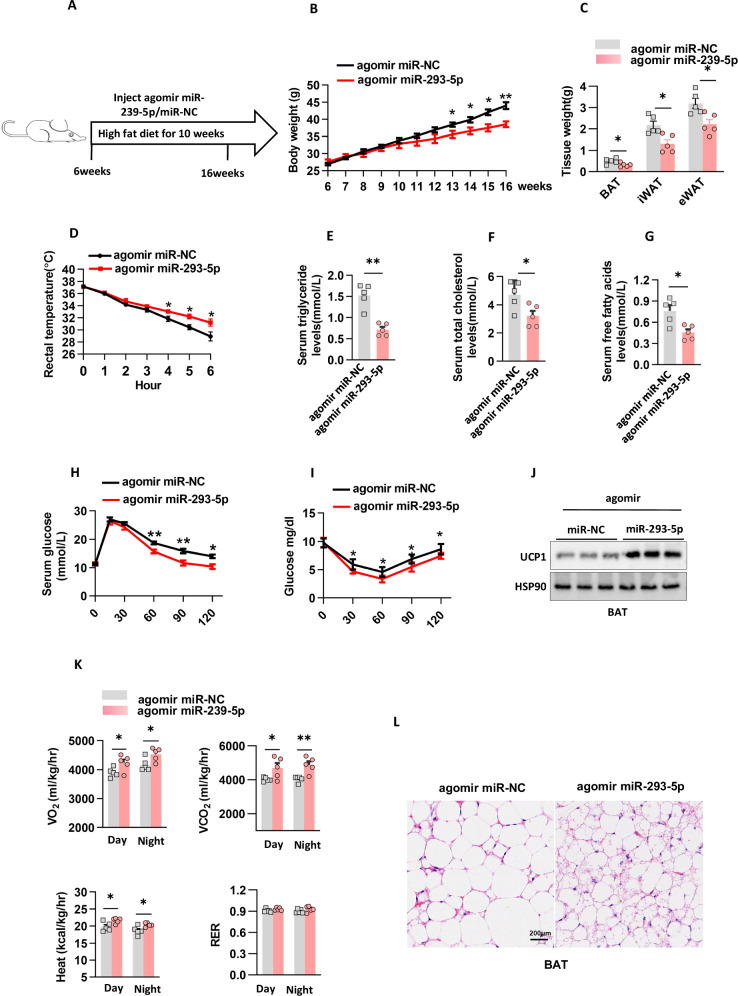
miR-293-5p therapy slowed obesity in mice. **A** Male C57BL/6 J mice were administered a high-fat diet and subjected to a protocol involving tail vein injection of either agomir miR-293-5p or miR-NC. **B** The body weight of the mice described in (**A**) was recorded, with each group comprising n = 5 mice. **C** The fat mass of the mice in (**A**) was measured, with each group consisting of n = 5 mice. **D** Rectal temperature was assessed in the mice from (**A**) when exposed to a 4°C environment, with n = 5 mice per group. **E**-**G**. Serum concentrations of triglycerides (**E**), total cholesterol (**F**), and free fatty acids (**G**) were evaluated in the mice from (**A**), with n = 5 mice per group. **H** A glucose tolerance test was conducted following the high-fat diet regimen in the mice from (**A**), with n = 5 mice per group. **I** An insulin tolerance test was performed subsequent to high-fat diet feeding in the mice from (**A**), with n = 5 mice per group. **J** UCP1 protein levels were quantified in BAT through n = 3 independent experiments. **K** Male C57BL/6 J mice underwent indirect calorimetry analysis under basal conditions, with each group containing n = 5 mice. **L** Hematoxylin and eosin (H&E) staining was performed on samples from the mice in (**A**) across different treatment groups, with a scale bar of 0.2 mm for BAT.

Previous research has demonstrated that obesity-induced dysfunction in β3-adrenergic signaling pathways impairs the thermogenic capacity of brown and beige adipocytes [19, 20]. To investigate the therapeutic potential of miR-293-5p in restoring adipose tissue responsiveness under obese conditions, we conducted a longitudinal intervention study using diet-induced obese mice. Over a period of 10 weeks of high-fat diet feeding, the animals received systemic administration of either agomir miR-293-5p or miR-NC via intravenous delivery (Fig. 6A). Although no differences were observed between groups in terms of caloric intake or hydration status (Supplementary Fig. 7A,B), a progressive divergence in body weight trajectories was noted, with agomir miR-293-5p-treated mice showing significant weight reduction from week 7 onward (Fig. 6B). Body composition analysis indicated that this anti-obesity effect was primarily due to substantial reductions in both inguinal and eWAT depots (Fig. 6C). The metabolic improvements extended beyond adiposity measures, as miR-293-5p-enhanced mice exhibited superior thermoregulatory capacity during cold challenge, evidenced by sustained higher core body temperatures (Fig. 6D).

The analysis of circulating metabolic parameters revealed a comprehensive improvement, characterized by significant reductions in triglyceride levels, total cholesterol, and free fatty acids compared to the control group (Fig. 6E–G). Functional assessments indicated enhanced whole-body metabolic flexibility, evidenced by improved glucose disposal rates and increased insulin sensitivity (Fig. 6H, I). Mechanistic investigations showed an upregulation of UCP1 protein expression in BAT, although this increase did not achieve statistical significance in iWAT (Fig. 6J; Supplementary Fig. 7D). Indirect calorimetry measurements supported these findings, demonstrating increased heat production along with elevated VO2 and VCO2 in the treatment group (Fig. 6K). Histopathological evaluation of BAT revealed a reduction in lipid droplet size and an enhanced multilocular morphology, indicative of activated thermogenic potential (Fig. 6L). Collectively, these multimodal data establish the administration of miR-293-5p as an effective strategy for mitigating diet-induced metabolic dysregulation through the coordinated enhancement of adipose tissue functionality and systemic energy expenditure.

### Tet1 (Ten-eleven translocation 1) functions as a direct target of miR-293-5p

**Fig. 7:**
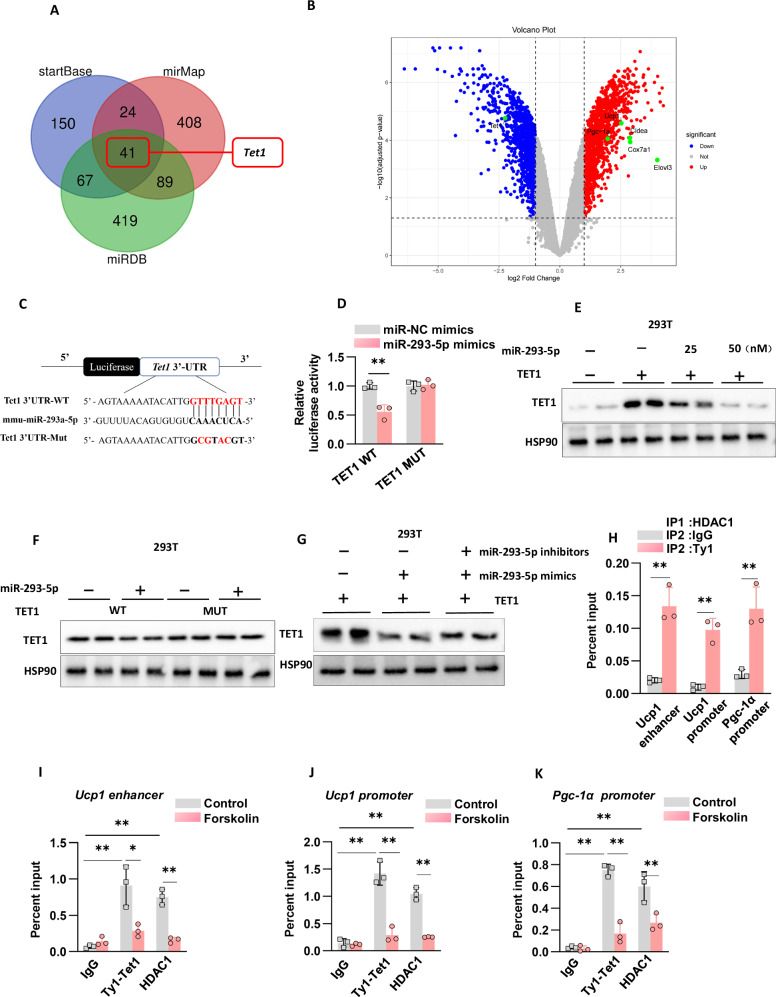
Tet1 (Ten-eleven translocation 1) functions as a direct target of miR-293-5p. **A** A Venn diagram was utilized to illustrate the target gene prediction algorithm, incorporating startBase, mirMap, and miRDB, to identify theoretical target genes of miR-293-5p. **B** The miRNA-seq analysis was conducted on primary brown fat cells treated with control hepExos and 3-day cold hepExos, with n = 4 independent experiments. **C** The binding sites for miR-293-5p on the Tet1 3’ untranslated regions (3’UTRs) were identified. **D** The results of the luciferase reporter assay demonstrated the interaction between miR-293-5p and Tet1 in 293 T cells co-transfected with either WT or mutant mouse Tet1, with n = 3 independent experiments. **E** Western blot analysis was performed to assess Tet1 and Myc protein levels in response to miR-293-5p mimics in 293 T cells co-transfected with WT Tet1. **F** Western blot analysis was also conducted to evaluate Tet1 and Myc protein levels in response to miR-293-5p mimics in 293 T cells co-transfected with either WT or mutant Tet1. **G** Western blot analysis of Tet1 protein level in response to the miR-293-5p mimics and miR-293-5p mimics plus inhibitors in 293 T cells cotransfected with WT Tet1. **H** ChIP-reChIP qPCR analysis was conducted on beige adipocytes transduced with either Ty1-Tet1 WT or GFP, utilizing HDAC1 for the first immunoprecipitation (IP), Ty1 for the second IP, and IgG as a control. The enrichment efficiency is expressed as a percentage of input at the specified binding sites, based on three independent experiments n = 3. **I**-**K** Ty1 and HDAC1 ChIP-qPCR analyses were performed on cells expressing Ty1-Tet1 WT, both with and without forskolin stimulation, across three independent experiments n = 3.

MicroRNAs mediate post-transcriptional regulation by engaging in complementary base pairing between their seed sequences and target mRNAs, resulting in translational inhibition or transcript degradation. To systematically identify targets of miR-293-5p, we employed an integrative approach combining computational predictions from three algorithms (TargetScan, miRmap, miRDB) with transcriptome profiling (Fig. 7A). This multi-platform strategy identified Tet1 as a high-confidence target, evidenced by a reduction in mRNA levels (Fig. 7B) and decreased protein expression following transfection with miR-293-5p mimics in adipocytes (Fig. 3F). The regulatory specificity was further validated through antisense inhibition experiments, where miR-293-5p inhibitors led to elevated TET1 protein levels (Fig. 3I). Cross-tissue validation in hepatocyte-adipocyte coculture systems demonstrated cell-nonautonomous regulation: adipocytes exposed to miR-293-5p-overexpressing hepatocytes showed reduced TET1 protein levels (Fig. 4F, I), whereas systems treated with miR-293-5p knockout or inhibitors exhibited increased TET1 expression (Fig. 4G, H).

In a mechanistic exploration, luciferase reporter assays conducted in 293 T cells revealed direct targeting, evidenced by a 45% suppression of wild-type TET1 3’UTR activity due to miR-293-5p binding, an effect that was nullified by mutations in the seed sequence (Fig. 7C, D). To confirm the direct interaction between miR-293-5p and TET1, we transfected 293 T cells with miR-293-5p mimics and TET1 mRNA. The introduction of miR-293-5p mimics led to a significant reduction in TET1 protein levels (Fig. 7E), an effect absent in cells expressing mutant TET1 protein (Fig. 7F). Furthermore, the application of miR-293-5p inhibitors effectively counteracted the miR-293-5p mimics, restoring TET1 protein expression (Fig. 7G). This dose-dependent, sequence-specific regulatory mechanism, which integrates gain-of-function suppression, loss-of-function rescue, and binding site mutagenesis, unequivocally establishes TET1 as a bona fide target of miR-293-5p, mediated by evolutionarily conserved seed-pairing interactions.

Recent evidence indicates that TET1 functions as a transcriptional repressor through cooperative interactions with various epigenetic regulators, including the Polycomb Repressive Complex 2 (PRC2), SIN3A, and histone deacetylases (HDACs), in diverse cellular environments. Building upon our previous finding that the inhibition of HDAC1, either through genetic manipulation or pharmacological intervention, significantly enhances the expression of UCP1/PGC-1α and oxidative metabolism [21], we proposed a functional interaction between TET1 and HDAC1 in the silencing of thermogenic genes. To investigate this hypothesis, we developed a system for the overexpression of TET1 tagged with a Ty1 epitope, thereby overcoming the limitations associated with the specificity of endogenous TET1 antibodies. Sequential chromatin immunoprecipitation (ChIP-reChIP) assays demonstrated significant co-occupancy of TET1 and HDAC1 at specific target loci (Fig. 7H). Notably, this co-localization was dynamically responsive to physiological stimuli; treatment with forskolin significantly reduced the occupancy of both TET1 and HDAC1 at these genomic regions (Fig. 7I–K), reflecting the known disassembly of repressive complexes during β-adrenergic receptor-activated thermogenesis.

### Overexpression of *Tet1* inhibits BAT thermogenic program in mice

**Fig. 8:**
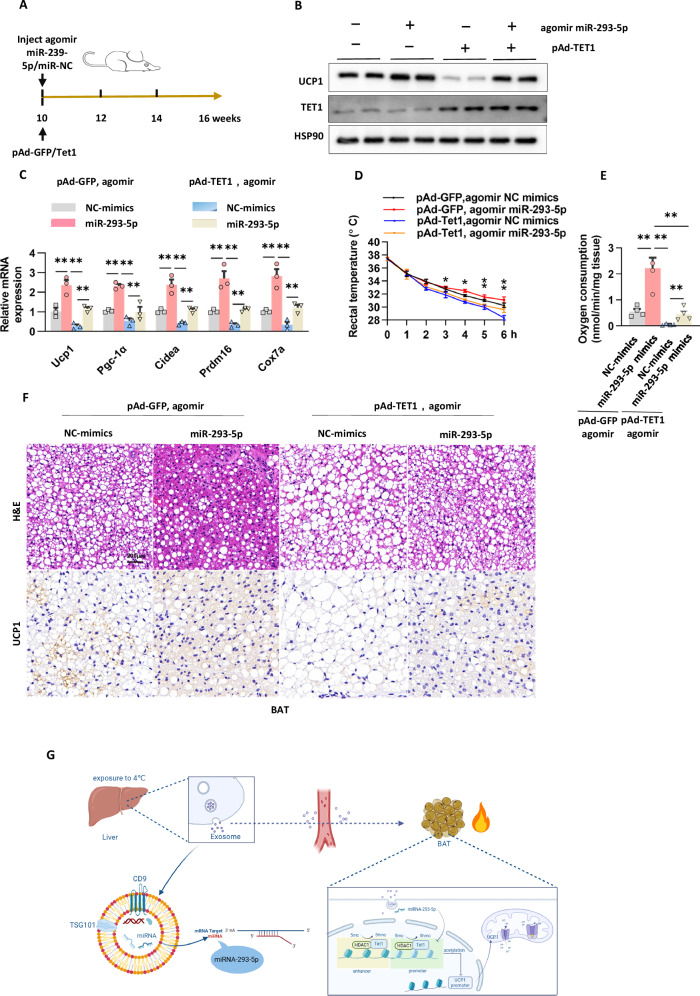
Overexpression of *Tet1* inhibits BAT thermogenic program in mice. **A** Description of the protocol for the injection of pAd-Tet1 and agomir miR-293-5p/NC in mice. **B** Analysis of Tet1 and UCP1 protein levels via Western blot in response to agomir miR-293-5p/NC injection in mice co-transfected with wild-type mouse pAd-TET1. **C** Quantitative PCR analysis of gene expression in BAT as described in (**A**), conducted over three independent experiments. **D** Measurement of core body temperature in mice from various treatment groups, based on five independent experiments. **E** Assessment of oxygen consumption rate in BAT using Clark electrodes, as outlined in (**A**), with data from four independent experiments. **F** Hematoxylin and eosin (H&E) staining and immunohistochemical detection of UCP1 protein expression in the BAT of mice from different treatment groups, with a scale bar of 0.2 mm. **G** Exposure to cold temperatures initiates the activation of the YBX1-dependent exosomal miRNA sorting mechanism in hepatocytes, leading to the selective enrichment of miR-293-5p within hepatic exosomes. These miRNA-enriched vesicles are then transported through systemic circulation to adipose tissue depots. In these depots, miR-293-5p specifically targets Tet1 mRNA by binding to its complementary 3’ untranslated region (3’UTR), thereby reducing Tet1-mediated epigenetic repression of thermogenic genes. This interorgan signaling cascade results in increased mitochondrial uncoupling through the upregulation of UCP1 and an enhanced capacity for oxidative phosphorylation, ultimately activating BAT-mediated energy expenditure.

To elucidate the hierarchical role of TET1 within miR-293-5p-regulated thermogenic pathways, we developed a BAT-specific TET1 gain-of-function model using adenoviral vector delivery (pAd-GFP *vs*. pAd-TET1) in conjunction with miR-293-5p agomir administration (Fig. 8A). Overexpression of TET1 significantly inhibited the activation of brown adipocytes, as evidenced by a reduction in UCP1 protein levels and the downregulation of key thermogenic regulators such as *Ucp1, Cox7a, Cideα, Pgc-1α*, and *Prdm16*, compared to GFP controls (Fig. 8B, C). These functional alterations resulted in diminished cold tolerance and a decreased mitochondrial oxygen consumption rate during cold exposure, phenotypes that were partially ameliorated by miR-293-5p agomir treatment (Fig. 8D, E). Histomorphometric analysis demonstrated TET1’s dual impact on lipid metabolism: adipocytes from miR-293-5p-treated mice exhibited smaller lipid droplets and increased UCP1 immunoreactivity, whereas TET1-overexpressing adipocytes showed hypertrophic lipid accumulation and reduced UCP1 signals (Fig. 8F).This epistatic relationship shows that TET1 functions downstream of miR-293-5p in a unique regulatory pathway, where overexpressing TET1 inhibits BAT activation by epigenetically silencing thermogenic enhancers.

## Discussion

Our study elucidates the role of hepatic exosomes (hepExos) as pivotal mediators in cold-adaptive thermogenesis through the systemic trafficking of miRNAs. A three-day exposure to cold conditions resulted in an increased secretion of hepatic exosomes, which were thermogenically primed and demonstrated the capacity to directly activate BAT by upregulating UCP1 and enhancing mitochondrial uncoupling. Through cross-tissue miRNA profiling and functional screening, we identified miR-293-5p as the central regulatory element, which was elevated in hepatocytes, circulating exosomes, and BAT under cold stress. Therapeutic agonism of miR-293-5p augmented cold-induced thermogenesis, leading to increased UCP1 protein expression in BAT and a reduction in visceral adiposity in diet-induced obese mice. This interorgan communication axis, facilitated by cold-inducible exosomal miRNA transfer, presents therapeutic potential for addressing metabolic syndrome by targeting and enhancing adaptive thermogenesis. The identification of miR-293-5p as both a biomarker and a functional effector in this pathway offers dual diagnostic and interventional strategies for obesity management.

Exosomes are generated through the endocytosis-mediated invagination of the plasma membrane. Substantial evidence indicates that exosomes serve as essential mediators of intracellular communication and intertissue crosstalk by delivering cargo to target cells and interacting with cell surface receptors [22, 23]. These vesicles encapsulate a diverse array of molecules, including proteins, mRNA, miRNA, and lncRNA, with miRNAs playing a pivotal role in exerting the majority of their biological effects [24, 25]. Specifically, adipocyte-derived exosomal miRNAs have been identified as key coordinators in the communication between adipocytes and other critical metabolic organs [8, 9]. Nevertheless, the metabolic regulatory capacity of hepatocyte-derived exosomal miRNAs in adipose tissue biology remains largely unexplored. To bridge this knowledge gap, we developed hepatic-specific YBX1 knockdown models, considering YBX1’s recognized function in facilitating miRNA loading into exosomes. In the context of hepatic YBX1 deficiency, there was a notable reduction in exosomal miRNA content, which coincided with impaired activation of BAT in response to cold exposure. Through small RNA sequencing of hepatocyte-derived exosomes from cold-adapted wild-type mice, miR-293-5p was identified as the predominant miRNA responsive to cold conditions. Importantly, miR-293-5p was the sole miRNA demonstrating multi-compartment synchronization, with upregulation observed in hepatocyte-derived exosomes, circulating exosomes, and BAT exosomes upon cold exposure. Validation via qRT-PCR indicated a strict spatial regulation, where hepatic exosomal miR-293-5p levels increased compared to controls, whereas BAT-resident miR-293-5p levels remained unchanged. This compartment-specific amplification supports the existence of a cold-inducible secretory mechanism rather than autonomous production within adipose tissue. The observed endocrine pathway, characterized by hepatic secretion, systemic circulation, and subsequent BAT uptake, provides mechanistic evidence for an exosome-mediated liver-adipose signaling axis that regulates adaptive thermogenesis.

Functional validation experiments have identified miR-293-5p as the primary mediator of thermogenesis facilitated by hepatic exosomes. In vitro experiments involving the administration of miR-293-5p mimics to differentiated 3T3-L1 adipocytes resulted in the specific upregulation of thermogenic markers, while adipogenic differentiation markers remained unaffected, thereby confirming the pathway-specific activation. The hepatic exosomal origin of this regulatory mechanism was substantiated by the predominance of miR-293-5p within hepatocyte-derived EVs. Systemic administration of agomir miR-293-5p in mice with diet-induced obesity ameliorated metabolic dysfunction by improving glucose disposal and insulin sensitivity. The dual metabolic effects of miR-293-5p are attributed to its role in promoting thermogenic reprogramming in BAT and inducing browning in WAT, collectively enhancing systemic energy expenditure. These coordinated adaptations across multiple tissues underscore miR-293-5p’s function as a master regulator of interorgan metabolic homeostasis via exosome-mediated epigenetic regulation.

The TET dioxygenase family, consisting of TET1, TET2, and TET3, plays a crucial role in epigenetic regulation by catalyzing the iterative oxidation of 5-methylcytosine (5mC) to 5-hydroxymethylcytosine (5hmC) and its subsequent derivatives, thereby dynamically modulating DNA methylation landscapes [26, 27]. In addition to their well-established functions in developmental processes, dysregulation of TET enzymes contributes to the pathogenesis of hematologic malignancies [28, 29]. Our study identifies a novel metabolic regulatory axis wherein miR-293-5p directly targets TET1, leading to a 45% reduction in its expression in brown adipocytes, as evidenced by dual-luciferase assays comparing wild-type and seed sequence-mutated TET1 3’UTR constructs. This repression mediated by miRNA is fully reversible, with locked nucleic acid (LNA) inhibitors capable of restoring TET1 expression. Mechanistically, TET1 collaborates with HDAC1 to enforce epigenetic silencing at thermogenic loci. In mice, adipose-specific ablation of TET1 enhances energy expenditure and imparts metabolic resilience, resulting in reduced weight gain induced by a high-fat diet and improved glucose tolerance [21]. Paradoxically, TET1 facilitates brown adipogenesis through the demethylation of the Prdm16 promoter [30], yet simultaneously inhibits thermogenesis in mature adipocytes via the deacetylation of PPARγ. This dual role is reminiscent of AMPKα1-deficient models, where the depletion of α-KG hinders TET-mediated activation of Prdm16, resulting in BAT dysfunction [30]. This bimodal regulatory framework—promoting adipogenesis while inhibiting thermogenesis—mirrors the dual functionality of Zfp423. Although ZFP423 is crucial for the commitment of white adipocytes [31], it also limits the beigeing process by sequestering EBF2 coactivators and diminishing PPARγ occupancy at UCP1 enhancers [32]. Similarly, the context-dependent effects of TET1—enhancing adipocyte differentiation while restricting thermogenic capacity—underscore the presence of epigenetic “brakes” that could be targeted therapeutically to decouple adipogenesis from metabolic dysfunction.

This study is subject to several limitations. Firstly, liver tissue comprises a diverse array of cell types, including hepatocytes, immune cells, and endothelial cells. This research specifically targets exosomes derived from hepatocytes, which constitute a significant proportion of extracellular vesicles entering the bloodstream from these cells. However, future investigations should incorporate exosomes isolated from liver non-parenchymal cells (NPCs) to address potential implications for this study. Secondly, our research concentrated on miRNAs, identifying miR-293-5p as a pivotal miRNA influencing BAT metabolism by comparing miRNA profiles in mice exposed to cold for three days. It is important to note that exosomal cargoes other than miRNAs may also play a role in mediating the effects of hepExos on lipid metabolism. To this end, we conducted additional experiments to profile the protein content of hepExos, identifying several protein candidates with significantly altered levels. The functional roles of these candidate proteins are currently under validation (unpublished data). Thirdly, the research was conducted exclusively using in vitro and in vivo mouse models, and validation with human data is presently lacking. Moreover, the animal experiments employed supra-physiological doses of miR-293-5p mimics and pAd-TET1. To validate these findings and conclusions, subsequent studies should incorporate adipocyte-specific overexpression of TET1 in murine models.

In summary, our research elucidates a regulatory axis between hepatocytes and brown adipose tissue, wherein cold-induced exosomal miR-293-5p inhibits TET1, thereby activating thermogenesis and promoting lipid oxidation and glucose disposal. Modulating the biogenesis of hepatic miR-293-5p presents innovative therapeutic opportunities for the treatment of metabolic disorders.

## Materials and methods

### Cell lines

Immortalized brown adipocytes, primary brown adipocytes, and beige adipocytes were cultured and differentiated according to established protocols [33–35]. Briefly, on day 0, confluent brown adipocytes were induced to differentiate using DMEM supplemented with 10% FBS, 20 nM insulin, 1 nM T3, 0.5 μM dexamethasone, 0.5 mM isobutylmethylxanthine, and 0.125 mM indomethacin for 48 hours. On day 2, the medium was changed to DMEM containing 10% FBS and 20 nM insulin, with 1 nM T3, for an additional 4 days to complete the differentiation process.

### Animal studies

All animal experiments were approved by the Binzhou Medical University Hospital Animal Care and Use Committee. Male C57BL6 mice, aged 6-8 weeks, were purchased from Jinan Pengyue Laboratory Animal Breeding Co., Ltd., Jinan, China. The mice were housed under a 12 h light and 12 h dark cycle at 22 °C with ad libitum access to water and a standard rodent chow diet, unless otherwise specified.

For 3-day cold exposure protocol, empty mouse cages are acclimatized in a 4 °C constant temperature chamber for 24 h prior to experimentation. Laboratory mice are subsequently housed in the pre-cooled 4 °C chamber for a continuous 72 h period.

An HFD, consisting of 60% of calories from fat, was obtained from Research Diets. HFD feeding commenced when the mice were 6 weeks old. Acute cold exposure was applied after 16 weeks of HFD feeding. To assess glucose tolerance and insulin sensitivity, mice underwent glucose tolerance tests (GTT) and insulin tolerance tests (ITT) following HFD. Mice were fasted for 16 h before GTT or 5 h before ITT, and then administered intraperitoneal injections of glucose (2 g/kg body mass) or insulin (0.75U kg^−1^ body mass), respectively. For indirect calorimetry, mice were acclimated in the Columbus Instruments Comprehensive Lab Animal Monitoring System, which measured energy expenditure and respiration under basal conditions. A scrambled agomiR was used as a control. The sequence for miR-293-5p agomiR is ACUCAAACUGUGUGACAUUUUG. For overexpression TET1, mice were injected bilaterally into the BATs with pAd-TET1 or pAd-GFP at a dose of 2 × 10^9^ PUF/kg in 0.1 mL saline once a week for 1 month.

### Isolation of primary hepatocytes

Mice underwent in situ hepatic perfusion initiated with calcium/magnesium-free HEPES-buffered solution A (10 mM HEPES, 1 mM glucose, 0.2 μM EGTA, 0.2% BSA, pH 7.4) via the inferior vena cava for 3–5 min until hepatic decolorization (beige parenchyma). Following this, the perfusate was switched to digestion buffer B (PBS supplemented with 1 mM CaCl_2_, 1 mM MgCl_2_, 0.5 mg/mL collagenase D, 30 mM HEPES, and 0.2% BSA). Perfusion was discontinued upon observable capsular disruption, after which the liver was rapidly excised into ice-cold buffer A. The digested hepatic tissue was mechanically dissociated in buffer A, filtered through a 100 μm nylon mesh, and subjected to low-speed centrifugation (60 × *g*, 4 °C, 6 min) to pellet parenchymal cells. The pellet underwent two washes with enzyme-free buffer B, followed by density gradient purification using 36% (v/v) Percoll™ (100 × *g*, 4 °C, 10 min). After aspirating the supernatant, the hepatocyte-enriched fraction was resuspended in buffer B and plated onto collagen-coated culture plates with Williams’ Medium E containing 10% heat-inactivated fetal bovine serum and 1× antibiotic-antimycotic cocktail. A medium replacement protocol was executed after 16 h of primary culture to remove non-adherent debris.

### Isolation and characterization of hepatocyte derived exosomes

Primary hepatocytes were maintained in exosome-depleted DMEM (supplemented with 10% FBS pre-cleared by 120,000 × *g* centrifugation for 16 h) for 6 h under standard culture conditions (37 °C, 5% CO_2_). Following this acclimatization phase, the medium was replaced with fresh exosome-free DMEM for a 36-h secretory period. Cellular supernatants (20 mL pooled from 5 × 10^6^ cells) were processed through differential ultracentrifugation: initial clarification at 3000 × *g* for 15 min (4 °C) to remove cellular debris, followed by filtration through 0.22 μm pore-size polyethersulfone membranes to eliminate microvesicles >220 nm. The filtrate underwent ultracentrifugation at 120,000 × *g* (4 °C, 60 min) using a Type 70 Ti rotor, with the resulting exosome pellet washed in ice-cold PBS and re-centrifuged under identical parameters to remove soluble protein contaminant. Exosome integrity was verified by transmission electron microscopy following negative staining with 2% uranyl acetate, revealing characteristic cup-shaped morphology with diameters of 30-150 nm. Exosome morphology was assessed using transmission electron microscopy at the Electron Microscopy Center of Binzhou Medical University.

### Isolation of plasma exosomes and exosomal miRNAs

Plasma specimens underwent initial clarification at 3000 × *g* (4 °C, 15 min) to eliminate cellular debris. Following rapid thaw at 37 °C, 250 μL aliquots were transferred to pre-chilled 1.5 mL low-binding tubes. Coagulation factors were removed by incubating supernatants with thromboplastin D (DiaPharma Group, 1:100 dilution) at 37 °C for 15 min, followed by a secondary clarification at 10,000 × *g* (RT, 5 min). Cleaned supernatants were processed for extracellular vesicle isolation using ExoQuick™ Exosome Precipitation Solution combined with RNase A to degrade free-floating RNA. The mixture was incubated overnight at 4 °C with gentle inversion. Prior to precipitation, murine RNase inhibitor was added to protect vesicular RNA integrity. EVs were pelleted at 1500 × *g* and washed twice with ice-cold PBS. Exosomal miRNA extraction was performed using the miRNeasy Micro Kit with on-column DNase I treatment, strictly adhering to MIQE guidelines. RNA integrity was verified via Bioanalyzer 2100 with RIN > 8.0, and quantified using a Qubit 4.0 Fluorometer.

### ChIP-reChIP

Primary immunocomplexes were eluted in 100 μL elution buffer (50 mM Tris pH 8.0, 1 mM EDTA, 1% SDS, 50 mM NaHCO3) at 65 °C for 10 min. Beads were treated with 40 μL 10 mM DTT at 37 °C for 30 min to dissociate residual antibodies. Combined eluates were diluted 10-fold in ChIP dilution buffer (16.7 mM Tris-HCl pH 8.0, 167 mM NaCl, 1.2 mM EDTA, 1.1% Triton X-100, 0.01% SDS) and subjected to secondary IP using anti-HDAC1 antibody under identical conditions.

### ChIP-qPCR

Cells were cross-linked with 1% formaldehyde for 10 min at 22-25°C. Chromatin was fragmented using a Covaris S220 Ultrasonicator (peak power: 140 W, duty factor: 5%, cycles/burst: 200) to generate 200-500 bp DNA fragments, verified by 2% agarose gel electrophoresis. Cleared lysates were divided into input controls and immunoprecipitation (IP) samples. IP samples were incubated overnight at 4°C with 5 μg of anti-Ty1 epitope antibody, anti-HDAC1, or species-matched IgG control. Protein-DNA complexes were captured by adding 20 μL pre-washed Dynabeads™ Protein G per reaction, followed by 1 h rotation at 4°C. Beads were successively washed in low-salt RIPA buffer (20 mM Tris-HCl [pH 8.0], 1 mM EDTA, 1% Triton x-100, 0.1% SDS, 140 mM NaCl, 0.1% Na deoxycholate), high-salt RIPA buffer (20 mM Tris-HCl [pH 8.0], 1 mM EDTA, 1% Triton x-100, 0.1% SDS, 500 mM NaCl, 0.1% Na deoxycholate), LiCl buffer (250 mM LiCl, 0.5% NP40, 0.5% Na deoxycholate, 1 mM EDTA, 10 mM Tris-HCl [pH 8.0]) and TE buffer (10 mM Tris-HCl [pH 8.0] and 1 mM EDTA). Crosslink reversal was performed in elution buffer (50 mM Tris-HCl pH 8.0, 1 mM EDTA, 100 mM NaCl, 0.5% SDS) containing 0.5 mg/mL proteinase K (Roche #03115887001) at 65 °C for 4 h. DNA was purified by phenol:chloroform:isoamyl alcohol (25:24:1 v/v) extraction, followed by ethanol precipitation (2.5 × volumes 100% ethanol, 0.1×volume 3 M NaOAc, −20 °C overnight). Real-time qPCR primers are listed in Table S2. All data were normalized to input.

### miRNA sequencing

Total RNA was extracted using TRIzol and stored in diethyl pyrocarbonate-treated water at −20 °C. miRNA libraries were prepared according to the manufacturer’s instructions using the NEBNext Illumina Small RNA Library Preparation Kit, which generates unique indexed libraries for each sample. Sequencing was performed on the Illumina HiSeq platform. The quality of the sequencing data associated with the sequencing core was assessed by the bioinformatics team.

### RNA-Seq Analysis

Total RNA was isolated from samples using the RNeasy Mini Kit (Qiagen, #74104), incorporating on-column DNase I digestion to remove genomic DNA contamination. RNA integrity was verified (RIN > 8.0) prior to library preparation. Strand-specific RNA-seq libraries were constructed using the BGI RNA Library Prep Kit following poly(A) + RNA selection, and sequenced (150 bp paired-end) on the BGISEQ-500 platform (average depth: 40 million reads/sample). Raw reads were quality-filtered and aligned to the mm10 genome (STAR v2.7.10a) using stringent parameters (“--outFilterMultimapNmax 1 --outSAMtype BAM SortedByCoordinate”). Mitochondrial RNA reads were excluded during alignment to prevent quantification bias. Gene-level counts were generated (featureCounts v2.0.1) against GENCODE M25 annotations. Differential expression analysis (edgeR v3.38.1) employed TMM normalization with significance thresholds (FDR < 0.05, |log2FC | >1). Co-expressed gene modules were identified through hierarchical clustering (Ward’s method; Pearson correlation distance). Functional enrichment of DEGs (FDR < 0.01) was performed using Enrichr (GO Biological Processes 2023; adjusted p < 0.05).

### miRNA quantitative PCR

The exosomal RNAs used were poly(A)-tailed with poly(A) polymerase, followed by reverse transcription using modified oligo(dT) primers and SMART MMLV reverse transcriptase. Small RNA assays with miRNA-specific primers were used to convert RNA from other sources into cDNA via stem-loop reverse transcription. Quantitative PCR was conducted using the Applied Biosystems 7500 system with SYBR Green mix and primers specific to each miRNA. The relative abundance of each miRNA was calculated after normalization to U6, and results were expressed as fold changes relative to the control group. The primer sequences are shown in Table S2.

### Mimics/inhibitors transfection

Commercially available miRNA mimics, inhibitors, and negative controls (RiboBio, Guangzhou, China) were used. Transfections were carried out using iMAX reagent (Invitrogen) with 50 nM miRNA mimics/inhibitors or negative controls, according to the manufacturer’s protocol. Brown preadipocytes and primary hepatic s were seeded in 12-well plates at an 80% confluence and transfected with 25 nM nonspecific RNA control or miRNA mimics/inhibitors. Cells were maintained in fresh medium 12 h post-transfection and subsequently harvested for Western blot or quantitative RNA analysis as required.

### Luciferase reporter gene assays

Various *Tet1* luciferase reporter plasmids (pTK81-IgK, 100 ng per transfection) were transiently transfected into HEK293 cells along with a Renilla luciferase vector (pRL, 10 ng per transfection). Cells were plated in 24-well plates and cultured for less than 24 h before transfection. Constructs of 100 ng pMIR REPORT containing wild-type or mutated miR-293-5p binding sequences were used. Luciferase activity was measured using the Dual-Luciferase Reporter Assay System (Promega) and normalized to Renilla luciferase activity. Each experiment was conducted in triplicate and independently repeated at least three times.

### Co-culture experiments

Co-culture experiments were performed using 12-well transwell plates with 0.4 μm pore-sized filters (Corning Costar, USA) for 24 h. Primary hepatic s were seeded in the transwell inserts, while brown preadipocytes were seeded in the bottom chamber.

### Oxygen Consumption

Freshly isolated adipose tissue deposits (approximately 40 mg) were minced in 1 mL phosphate-buffered saline supplemented with 25 mM glucose, 1 mM pyruvate, and 2% BSA. Cultured adipocytes were trypsinized, collected by centrifugation, and resuspended in the same buffer. Oxygen consumption was measured using a Clark electrode (Oxygraph+ System, Hansatech), and data were normalized according to tissue weight or total protein content.

### In vivo adeno-associated serotype 9 virus injection into BAT

Recombinant adeno-associated serotype 9 virus with TBG promoter for YBX1 knockdown (AAV9-shYbx1) was purchased from Hanbio Biotechnology Co (Shanghai, China). The virus was diluted with sterile 1× PBS. We used AAV9-Ctrl as control. Five microliters of either AAV9-shYbx1 or AAV9-Ctrl was delivered into the BAT (total virus: 2 × 10^11^VG, injection volume: 200 µL, administered once every four weeks).

### In vivo adenovirus injection into BAT

Adenoviruses were purified by cesium chloride ultracentrifugation, and virus titers were determined in HEK293T cells by counting GFP-positive cells. Two-month-old male mice were used for viral injection. 100 µL of each adenovirus (5 × 10^9^ pfu) diluted in phosphate-buffered saline was injected bilaterally into the BAT. Mice were injected every 3 weeks for a total of two injections.

### Adenoviral expression vectors

The adenoviral expression vector pAd/CMV/V5-DEST, encoding TET1, was constructed following the manufacturer’s protocol. The TET1 sequence was amplified using the following primers:

5’- TATGTCGACATGTCTCGGTCCCGCCCCGCAAA-3’ (forward) and 5’- TAATTCTAGATTAGACCCAACGATTGTAGGGT-3’ (reverse).

### Serum triglyceride, total cholesterol and free fatty acids levels

Serum triglyceride, total cholesterol, and free fatty acids levels were assessed using a triglyceride assay kit according to the manufacturer’s instructions.

### Oil red O staining

Mature adipocytes were washed once with phosphate-buffered saline and fixed in 4% buffered formaldehyde at room temperature. The cells were then stained with Oil Red O working solution for 1 hour. After staining, cells were washed several times with Milli-Q water and prepared for microscopic imaging.

### RNA preparation and quantitative real-time PCR

Total RNA from cells and tissues was isolated using TRIzol (Invitrogen). Reverse transcription was performed using HiScript® Q RT SuperMix (Vazyme) according to the manufacturer’s protocols. Quantitative real-time PCR (qPCR) was conducted using the ViiA 7 Real-Time PCR System (Applied Biosystems). The primer sequences are shown in Table S2.

### Western blot analysis

Cell or tissue samples were homogenized in cell lysis buffer [150 mM NaCl, 0.5% Triton-X-100, 5% glycerol, 50 mM Tris-HCl (pH 7.5), 1 mM PMSF, and a protease inhibitors mixture (Roche)]. Lysates were centrifuged at 13,000 g for 10 min at 4 °C. Supernatants were quantified for protein concentration and analyzed by Western blotting with the indicated antibodies. The antibody information is listed in Table S3.

### Hematoxylin-eosin staining and immunohistochemistry

Fresh tissue samples were fixed in 4% paraformaldehyde, embedded in paraffin, and stained with hematoxylin-eosin at room temperature for 24 h. Histological images were captured using a microscope. For immunohistochemistry, slides were blocked with 2% horse serum, incubated overnight at 4 °C with anti-UCP1 (1:150), and then washed. Slides were incubated with an appropriate peroxidase polymer-linked secondary antibody for 30 min, stained with 3,3’-diaminobenzidine substrate, and counterstained with hematoxylin.

### Data analysis

Data are presented as mean ± standard error of the mean (SEM). Differences between two groups were assessed using an unpaired two-tailed Student’s t-test. One-way analysis of variance (ANOVA) followed by Tukey’s test was used to evaluate the statistical significanceof three or more groups. Statistical significance is indicated as *p < 0.05, **p < 0.01, or ***p < 0.001.

## Supplementary information

supp figure

wb data

Table S1

## Data Availability

All data, methods, and results of statistical analyses are reported in this paper and the associated Supplementary Materials. Any specific inquiries can be addressed to the corresponding author.
