# Supplementary material for: Cold-induced hepatocyte-derived exosomes activate brown adipose thermogenesis via miR-293-5p-mediated transcriptional reprogramming
Source: Cell Death Discov. 2025 Aug 22;11:396. doi: 10.1038/s41420-025-02697-1 (PMC12373855; doi:10.1038/s41420-025-02697-1)
Supplement: Supplementary file 1 — supp figure [file 41420_2025_2697_MOESM1_ESM.pptx]

## Slide 1
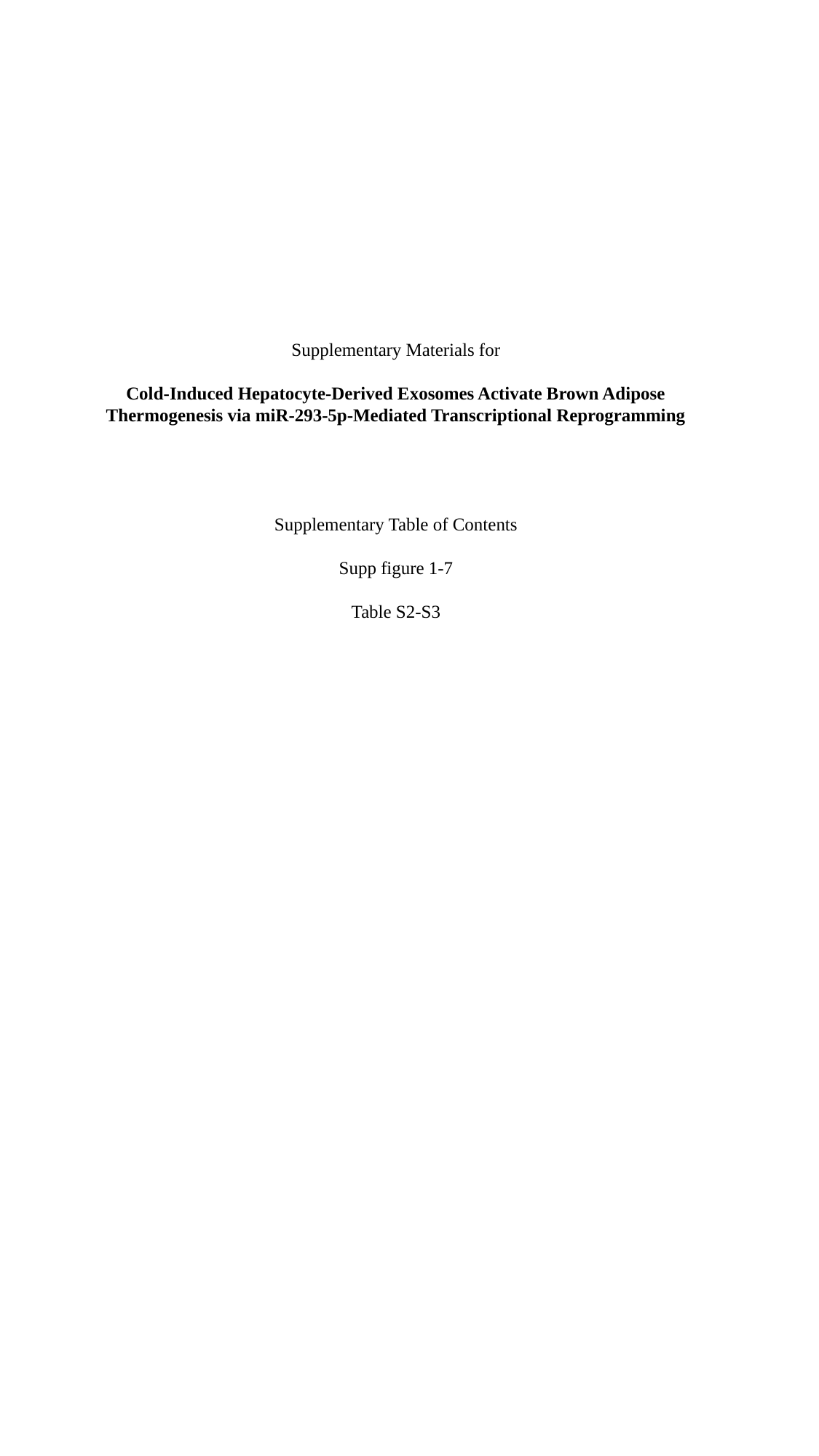

Supplementary Materials for
Cold-Induced Hepatocyte-Derived Exosomes Activate Brown Adipose Thermogenesis via miR-293-5p-Mediated Transcriptional Reprogramming
Supplementary Table of Contents
Supp figure 1-7
Table S2-S3

## Slide 2
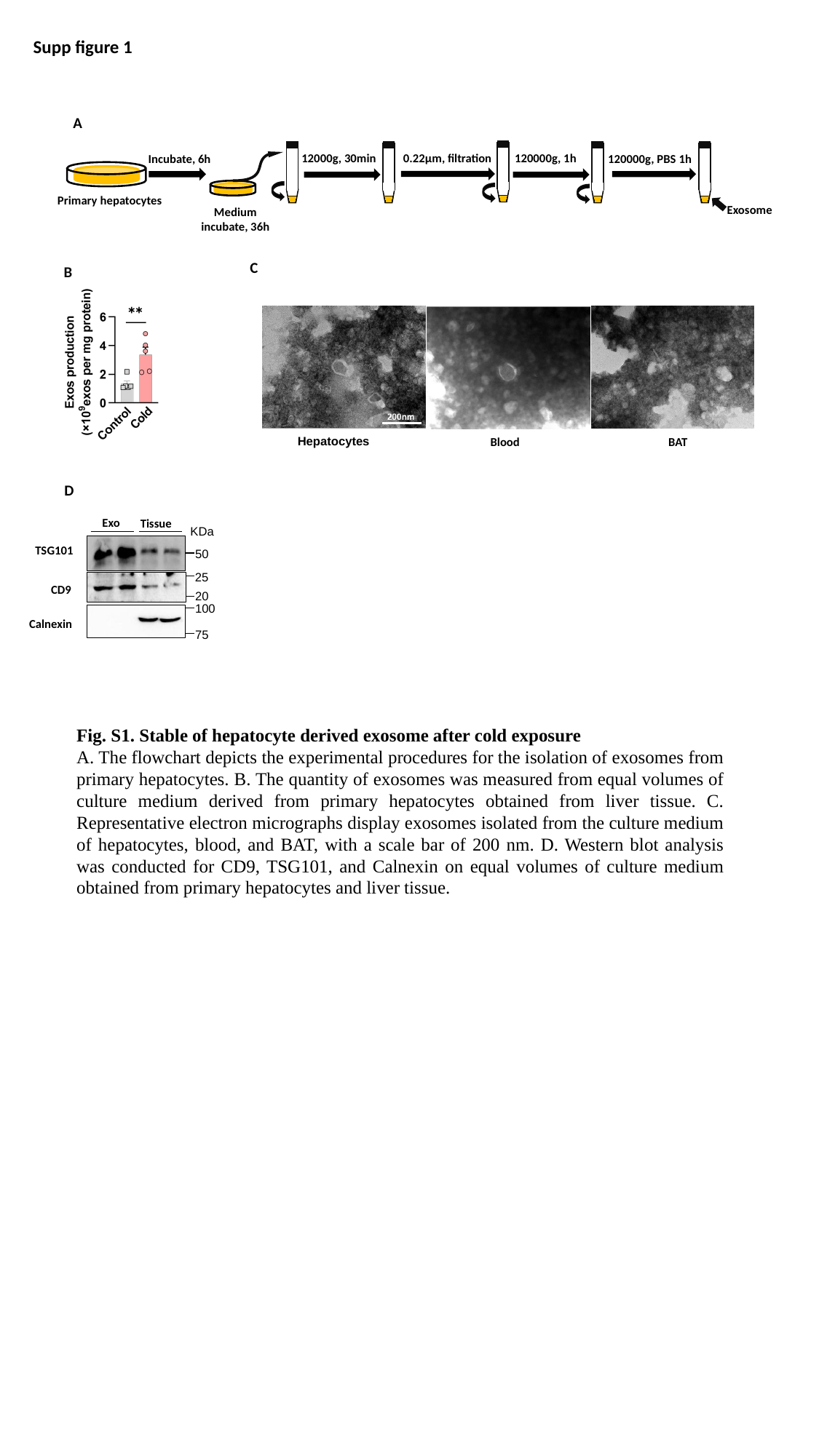

Supp figure 1
A
0.22μm, filtration
12000g, 30min
120000g, 1h
120000g, PBS 1h
Incubate, 6h
Primary hepatocytes
Medium incubate, 36h
 Exosome
C
Hepatocytes
Blood
BAT
B
D
Exo
Tissue
KDa
TSG101
50
25
CD9
20
100
Calnexin
75
Fig. S1. Stable of hepatocyte derived exosome after cold exposure
A. The flowchart depicts the experimental procedures for the isolation of exosomes from primary hepatocytes. B. The quantity of exosomes was measured from equal volumes of culture medium derived from primary hepatocytes obtained from liver tissue. C. Representative electron micrographs display exosomes isolated from the culture medium of hepatocytes, blood, and BAT, with a scale bar of 200 nm. D. Western blot analysis was conducted for CD9, TSG101, and Calnexin on equal volumes of culture medium obtained from primary hepatocytes and liver tissue.

## Slide 3
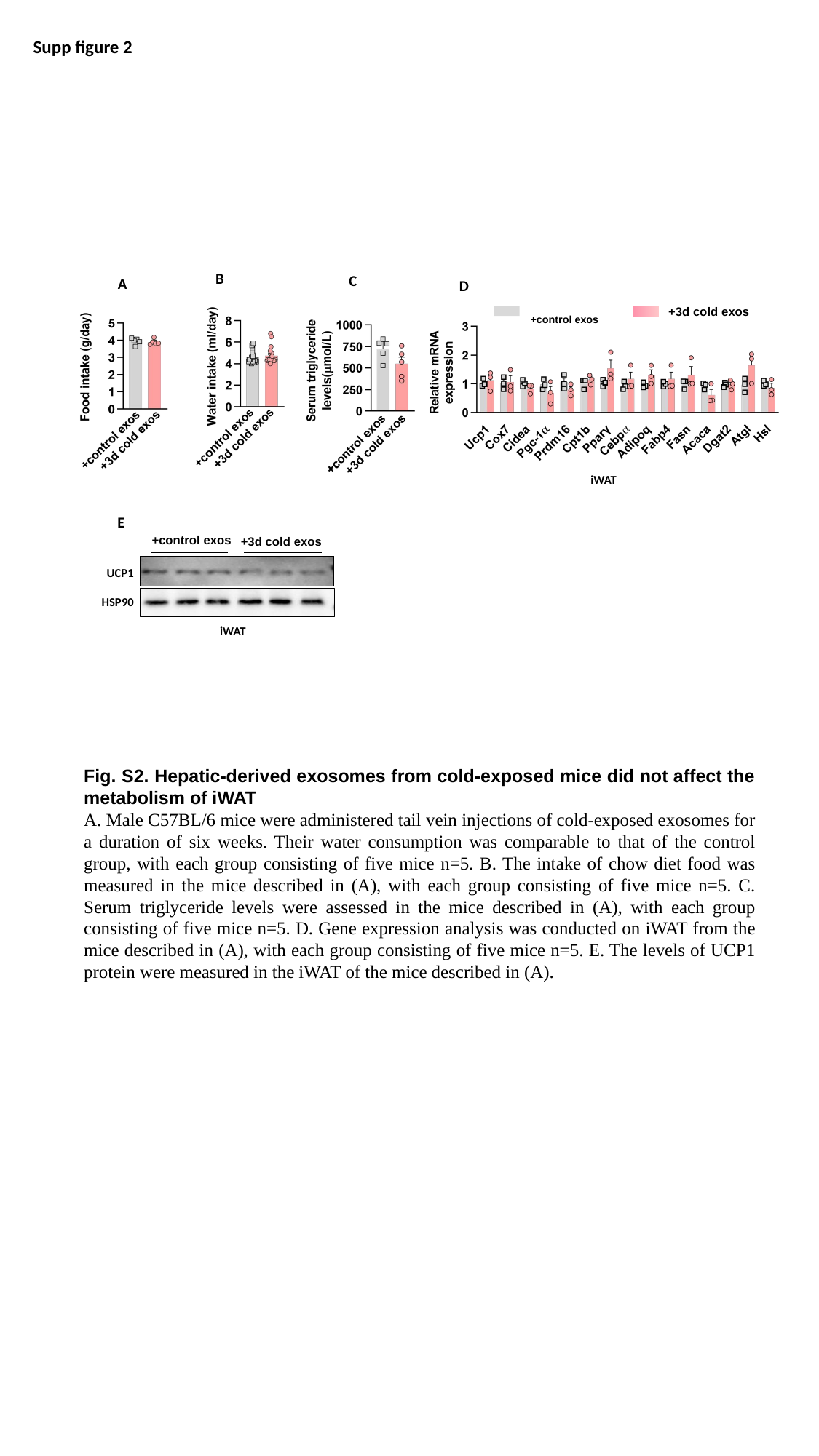

Supp figure 2
B
C
A
D
+control exos
+3d cold exos
iWAT
E
+control exos
+3d cold exos
UCP1
HSP90
iWAT
Fig. S2. Hepatic-derived exosomes from cold-exposed mice did not affect the metabolism of iWAT
A. Male C57BL/6 mice were administered tail vein injections of cold-exposed exosomes for a duration of six weeks. Their water consumption was comparable to that of the control group, with each group consisting of five mice n=5. B. The intake of chow diet food was measured in the mice described in (A), with each group consisting of five mice n=5. C. Serum triglyceride levels were assessed in the mice described in (A), with each group consisting of five mice n=5. D. Gene expression analysis was conducted on iWAT from the mice described in (A), with each group consisting of five mice n=5. E. The levels of UCP1 protein were measured in the iWAT of the mice described in (A).

## Slide 4
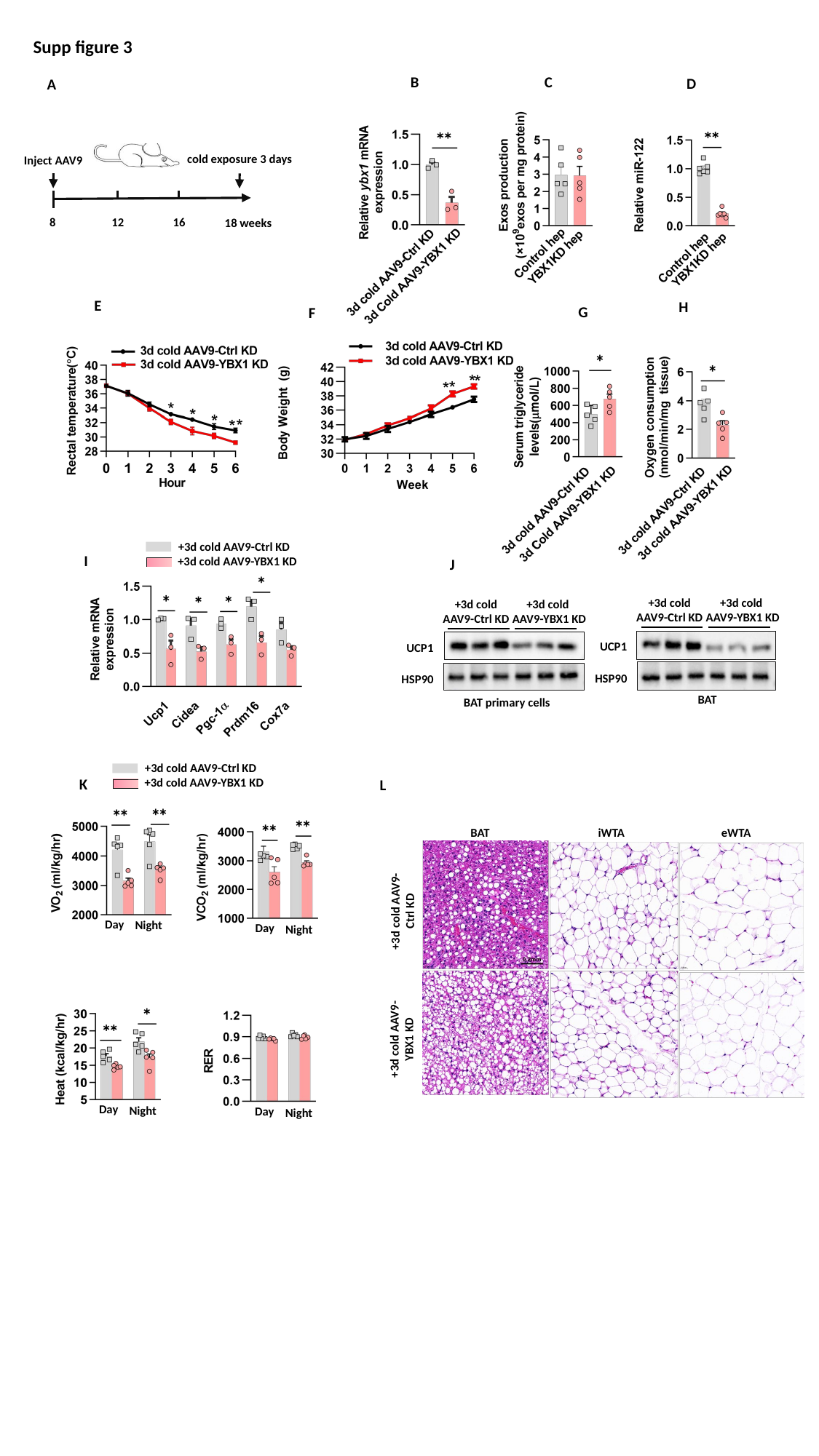

Supp figure 3
B
C
D
A
cold exposure 3 days
Inject AAV9
8
12
16
18 weeks
E
H
G
F
+3d cold AAV9-Ctrl KD
+3d cold AAV9-YBX1 KD
I
J
+3d cold
AAV9-YBX1 KD
+3d cold AAV9-Ctrl KD
UCP1
HSP90
BAT
+3d cold
AAV9-YBX1 KD
+3d cold AAV9-Ctrl KD
UCP1
HSP90
BAT primary cells
+3d cold AAV9-Ctrl KD
+3d cold AAV9-YBX1 KD
K
Day
Night
Day
Night
Day
Night
Day
Night
L
BAT
iWTA
eWTA
+3d cold AAV9-Ctrl KD
+3d cold AAV9-YBX1 KD

## Slide 5
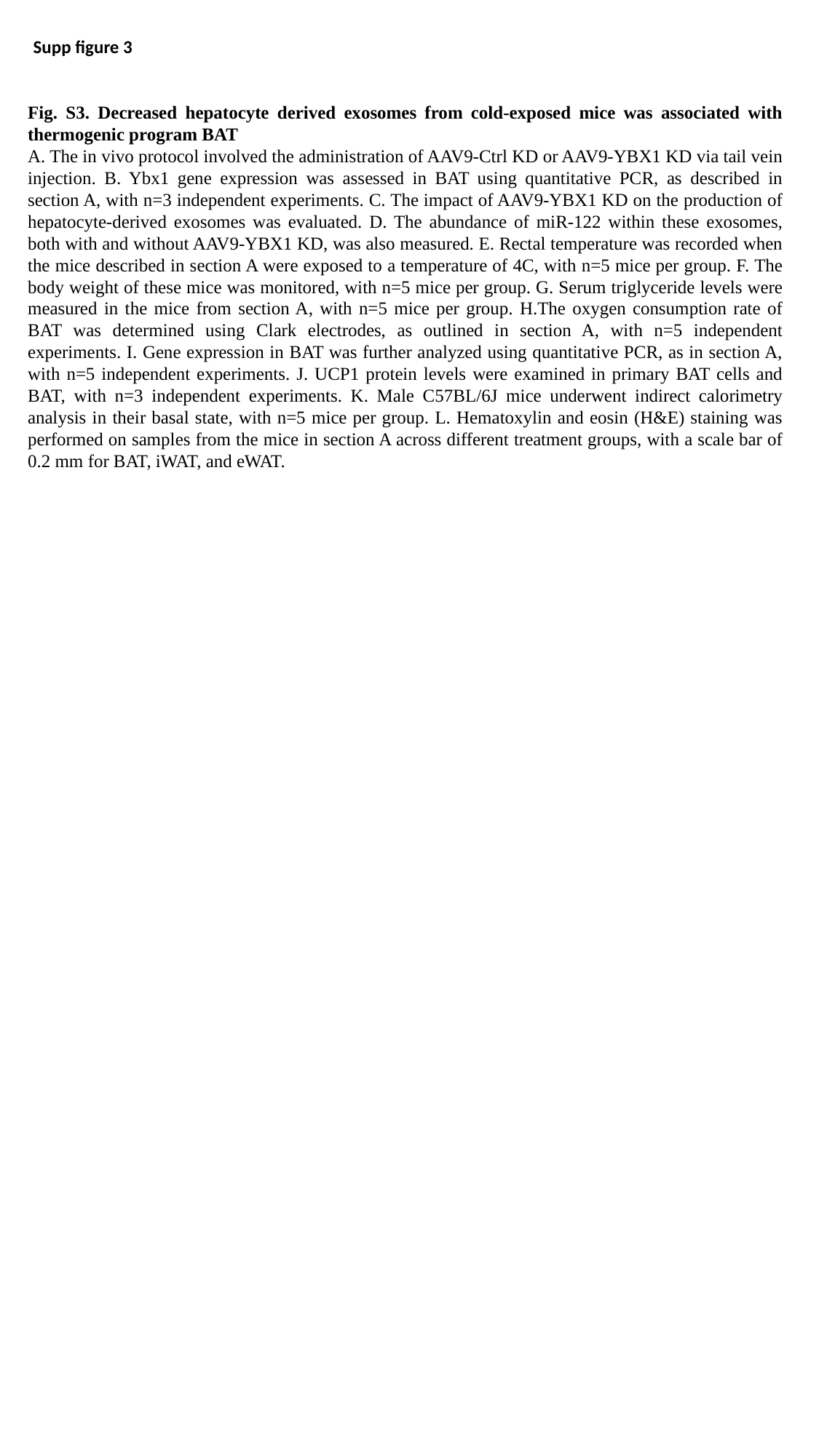

Supp figure 3
Fig. S3. Decreased hepatocyte derived exosomes from cold-exposed mice was associated with thermogenic program BAT
A. The in vivo protocol involved the administration of AAV9-Ctrl KD or AAV9-YBX1 KD via tail vein injection. B. Ybx1 gene expression was assessed in BAT using quantitative PCR, as described in section A, with n=3 independent experiments. C. The impact of AAV9-YBX1 KD on the production of hepatocyte-derived exosomes was evaluated. D. The abundance of miR-122 within these exosomes, both with and without AAV9-YBX1 KD, was also measured. E. Rectal temperature was recorded when the mice described in section A were exposed to a temperature of 4C, with n=5 mice per group. F. The body weight of these mice was monitored, with n=5 mice per group. G. Serum triglyceride levels were measured in the mice from section A, with n=5 mice per group. H.The oxygen consumption rate of BAT was determined using Clark electrodes, as outlined in section A, with n=5 independent experiments. I. Gene expression in BAT was further analyzed using quantitative PCR, as in section A, with n=5 independent experiments. J. UCP1 protein levels were examined in primary BAT cells and BAT, with n=3 independent experiments. K. Male C57BL/6J mice underwent indirect calorimetry analysis in their basal state, with n=5 mice per group. L. Hematoxylin and eosin (H&E) staining was performed on samples from the mice in section A across different treatment groups, with a scale bar of 0.2 mm for BAT, iWAT, and eWAT.

## Slide 6
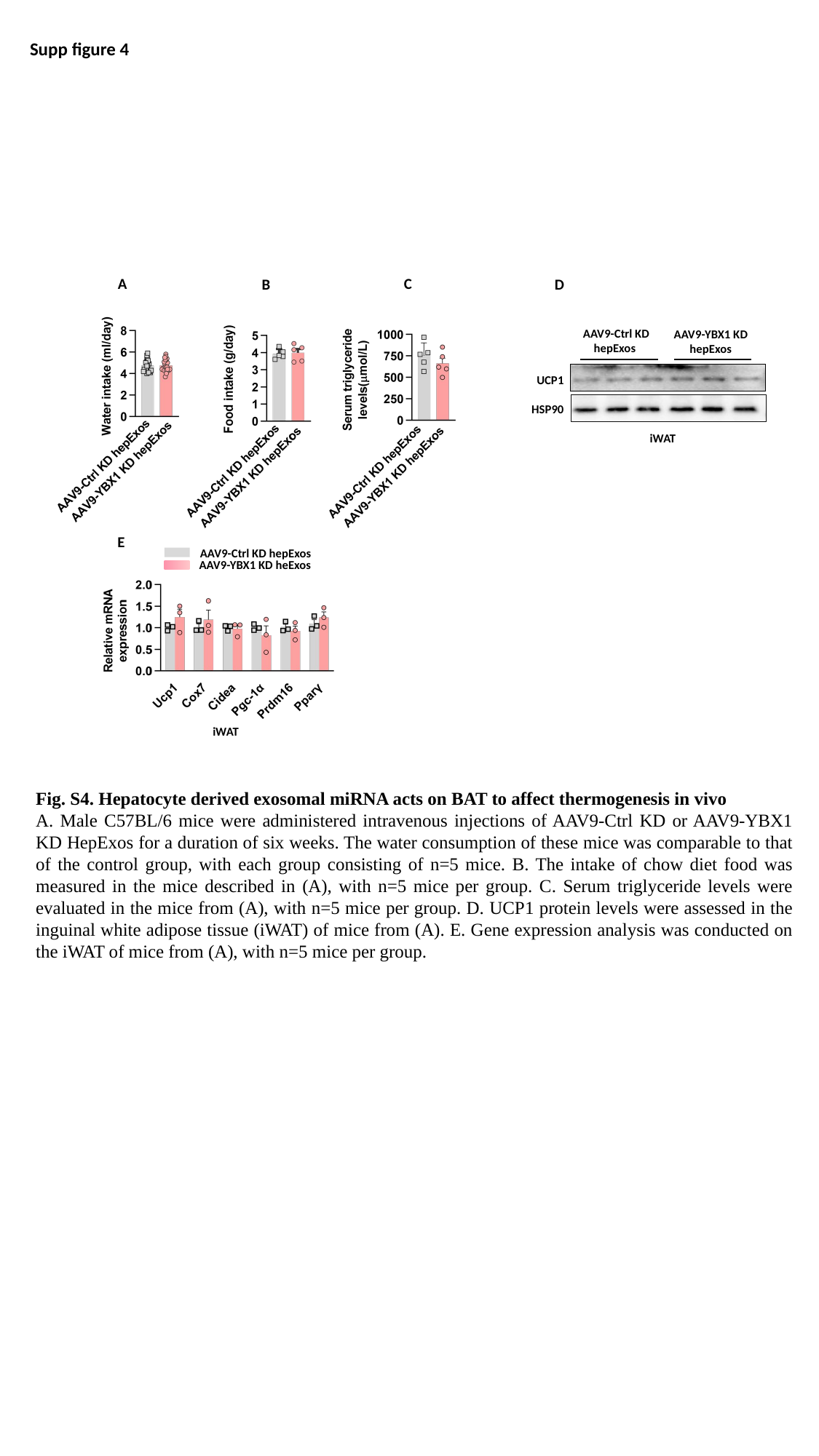

Supp figure 4
C
A
B
D
AAV9-Ctrl KD hepExos
AAV9-YBX1 KD
hepExos
UCP1
HSP90
iWAT
E
 AAV9-Ctrl KD hepExos
AAV9-YBX1 KD heExos
iWAT
Fig. S4. Hepatocyte derived exosomal miRNA acts on BAT to affect thermogenesis in vivo
A. Male C57BL/6 mice were administered intravenous injections of AAV9-Ctrl KD or AAV9-YBX1 KD HepExos for a duration of six weeks. The water consumption of these mice was comparable to that of the control group, with each group consisting of n=5 mice. B. The intake of chow diet food was measured in the mice described in (A), with n=5 mice per group. C. Serum triglyceride levels were evaluated in the mice from (A), with n=5 mice per group. D. UCP1 protein levels were assessed in the inguinal white adipose tissue (iWAT) of mice from (A). E. Gene expression analysis was conducted on the iWAT of mice from (A), with n=5 mice per group.

## Slide 7
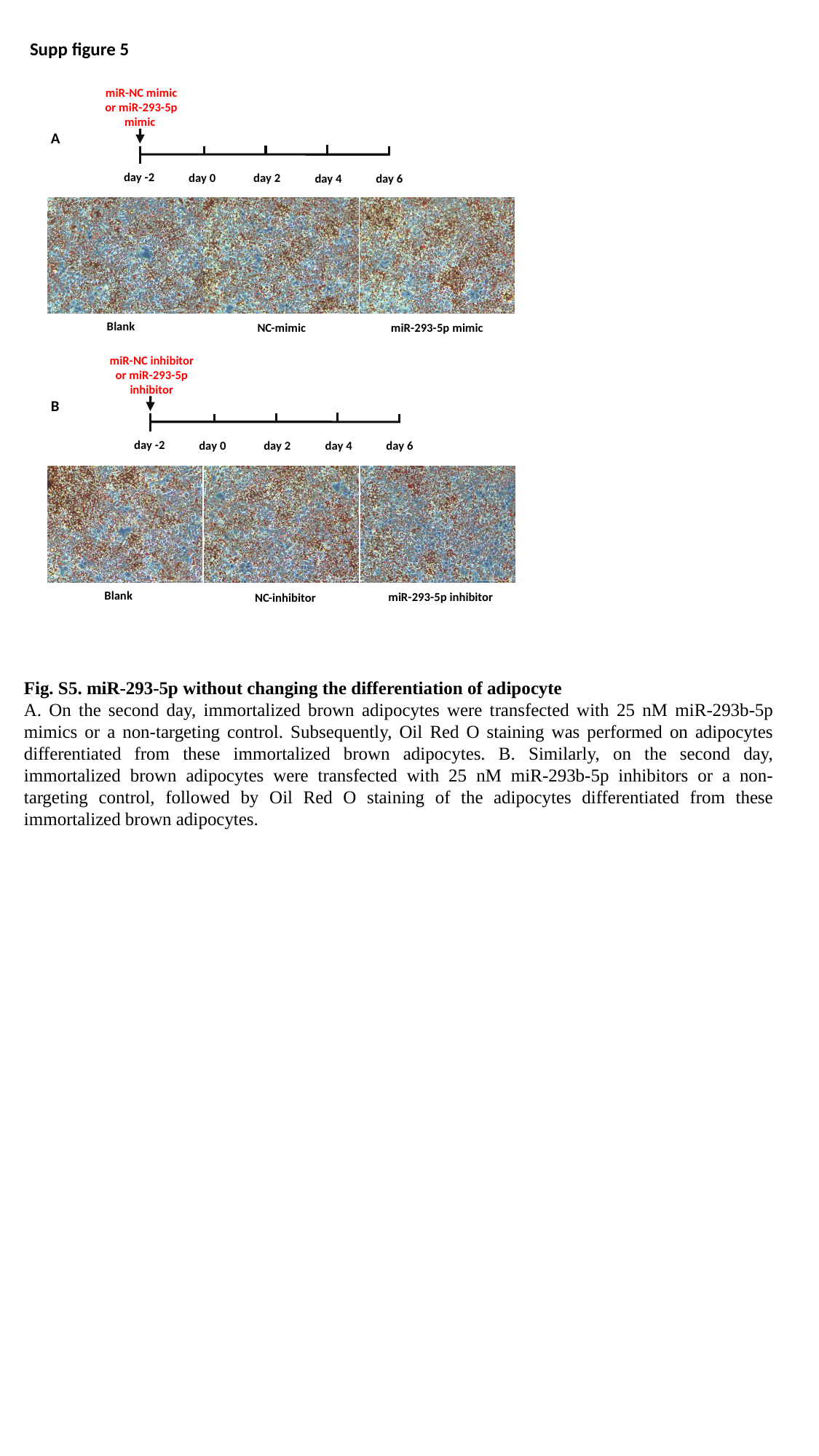

Supp figure 5
miR-NC mimic or miR-293-5p mimic
day -2
day 0
day 2
day 4
day 6
Blank
miR-293-5p mimic
NC-mimic
A
B
miR-NC inhibitor or miR-293-5p inhibitor
day -2
day 0
day 2
day 4
day 6
Blank
miR-293-5p inhibitor
NC-inhibitor
Fig. S5. miR-293-5p without changing the differentiation of adipocyte
A. On the second day, immortalized brown adipocytes were transfected with 25 nM miR-293b-5p mimics or a non-targeting control. Subsequently, Oil Red O staining was performed on adipocytes differentiated from these immortalized brown adipocytes. B. Similarly, on the second day, immortalized brown adipocytes were transfected with 25 nM miR-293b-5p inhibitors or a non-targeting control, followed by Oil Red O staining of the adipocytes differentiated from these immortalized brown adipocytes.

## Slide 8
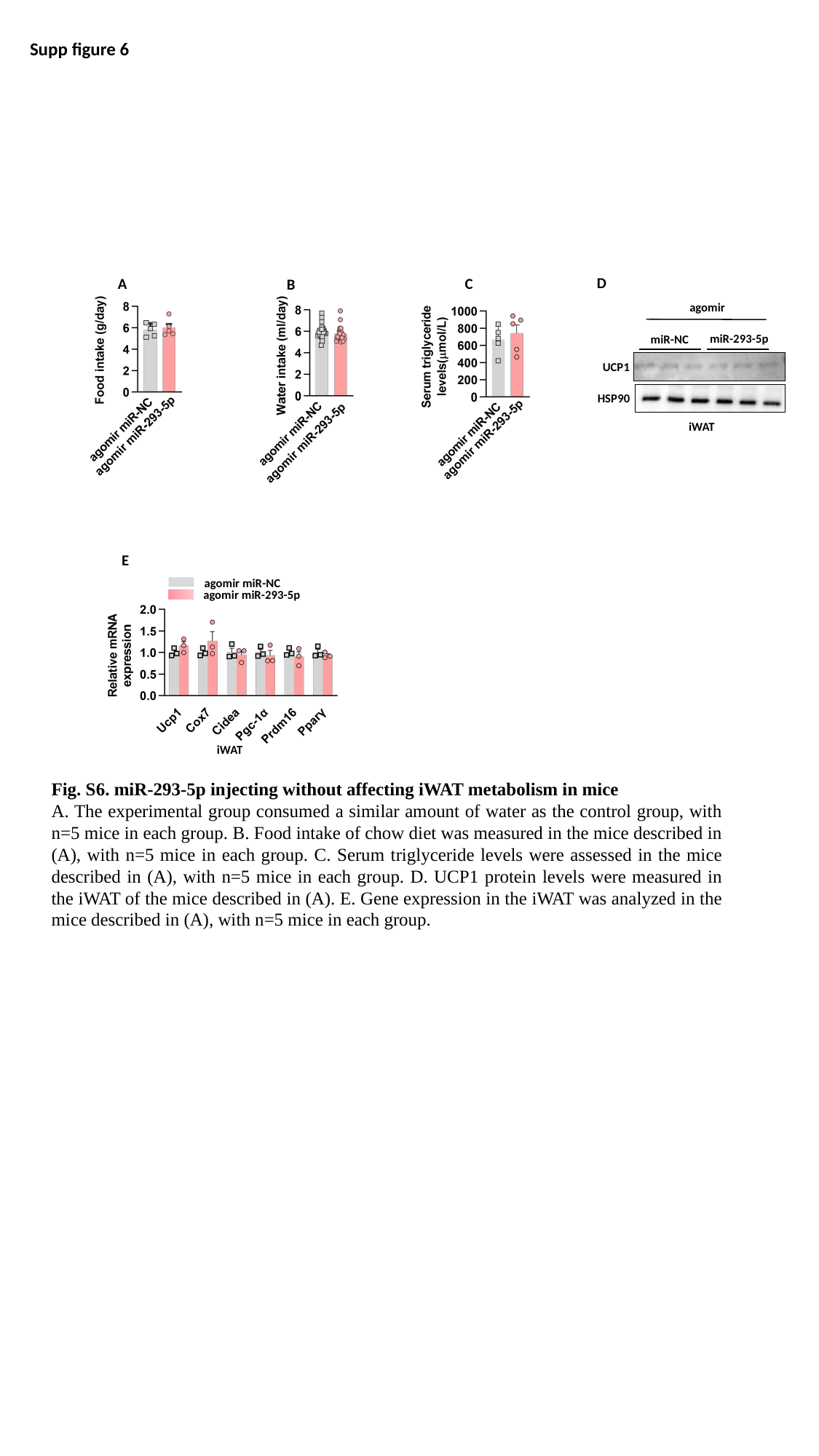

Supp figure 6
D
agomir
miR-293-5p
miR-NC
UCP1
HSP90
iWAT
C
A
B
E
 agomir miR-NC
agomir miR-293-5p
iWAT
Fig. S6. miR-293-5p injecting without affecting iWAT metabolism in mice
A. The experimental group consumed a similar amount of water as the control group, with n=5 mice in each group. B. Food intake of chow diet was measured in the mice described in (A), with n=5 mice in each group. C. Serum triglyceride levels were assessed in the mice described in (A), with n=5 mice in each group. D. UCP1 protein levels were measured in the iWAT of the mice described in (A). E. Gene expression in the iWAT was analyzed in the mice described in (A), with n=5 mice in each group.

## Slide 9
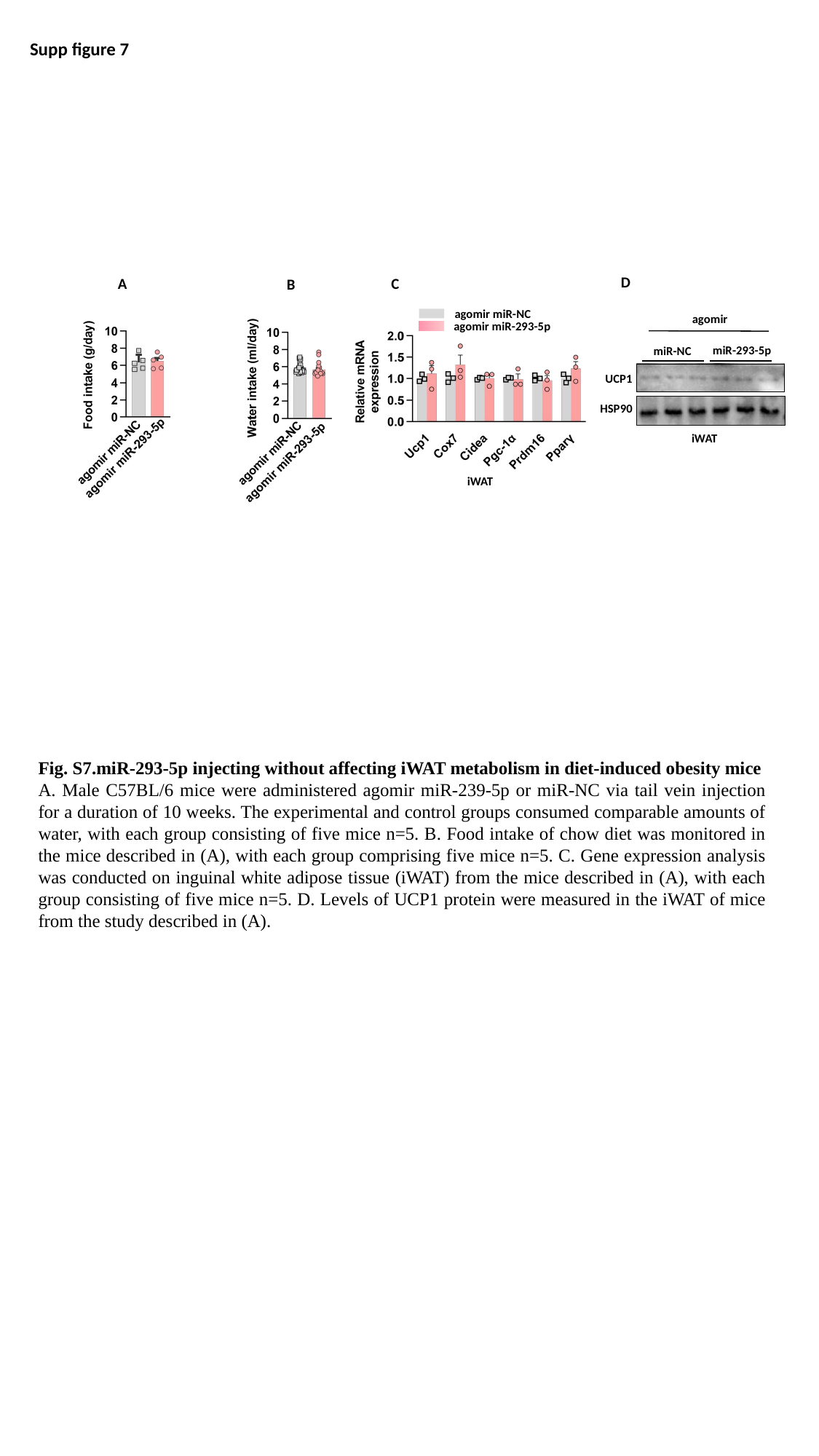

Supp figure 7
D
agomir
miR-293-5p
miR-NC
UCP1
HSP90
iWAT
C
 agomir miR-NC
agomir miR-293-5p
iWAT
A
B
Fig. S7.miR-293-5p injecting without affecting iWAT metabolism in diet-induced obesity mice
A. Male C57BL/6 mice were administered agomir miR-239-5p or miR-NC via tail vein injection for a duration of 10 weeks. The experimental and control groups consumed comparable amounts of water, with each group consisting of five mice n=5. B. Food intake of chow diet was monitored in the mice described in (A), with each group comprising five mice n=5. C. Gene expression analysis was conducted on inguinal white adipose tissue (iWAT) from the mice described in (A), with each group consisting of five mice n=5. D. Levels of UCP1 protein were measured in the iWAT of mice from the study described in (A).

## Slide 10
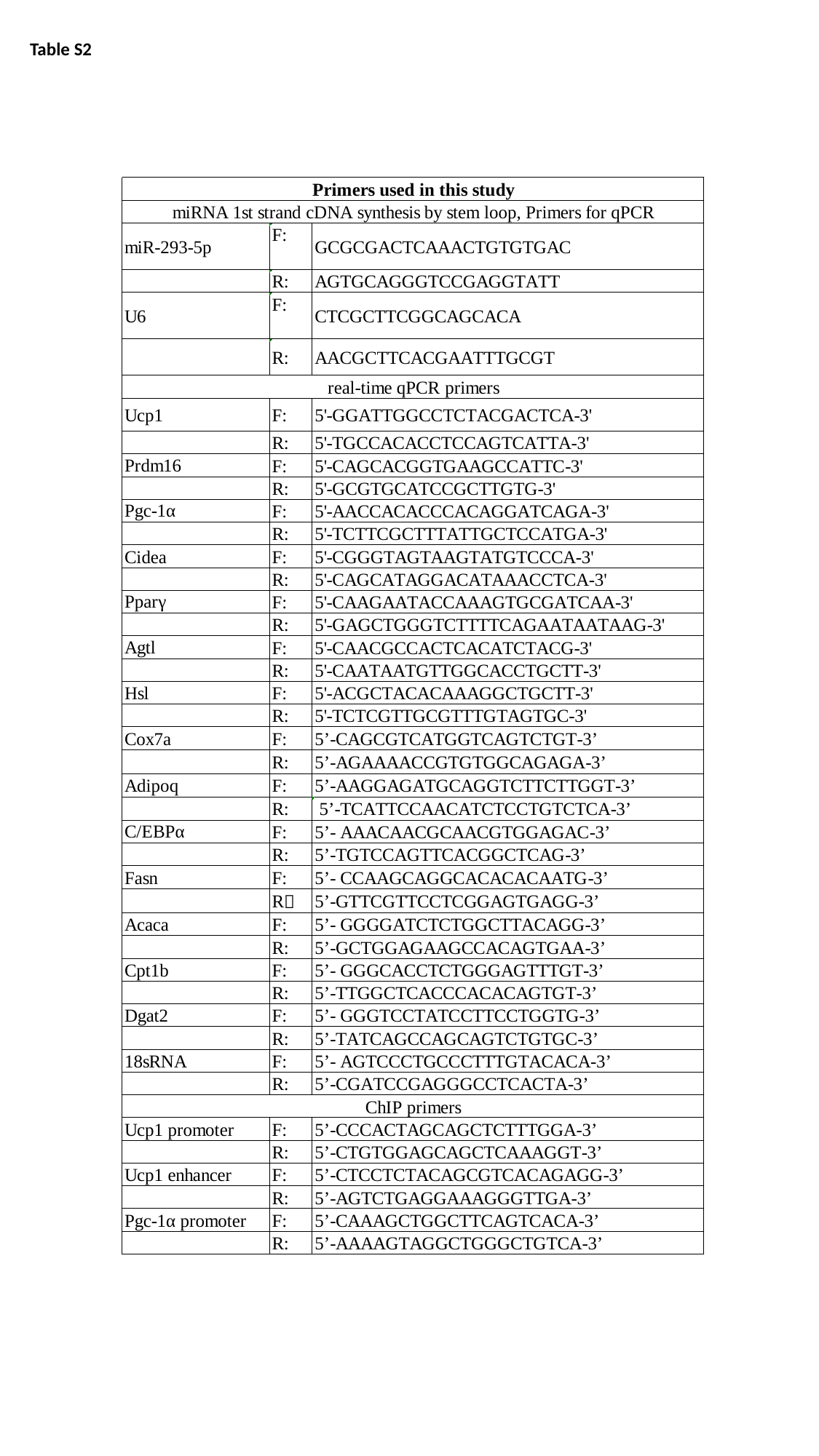

Table S2

## Slide 11
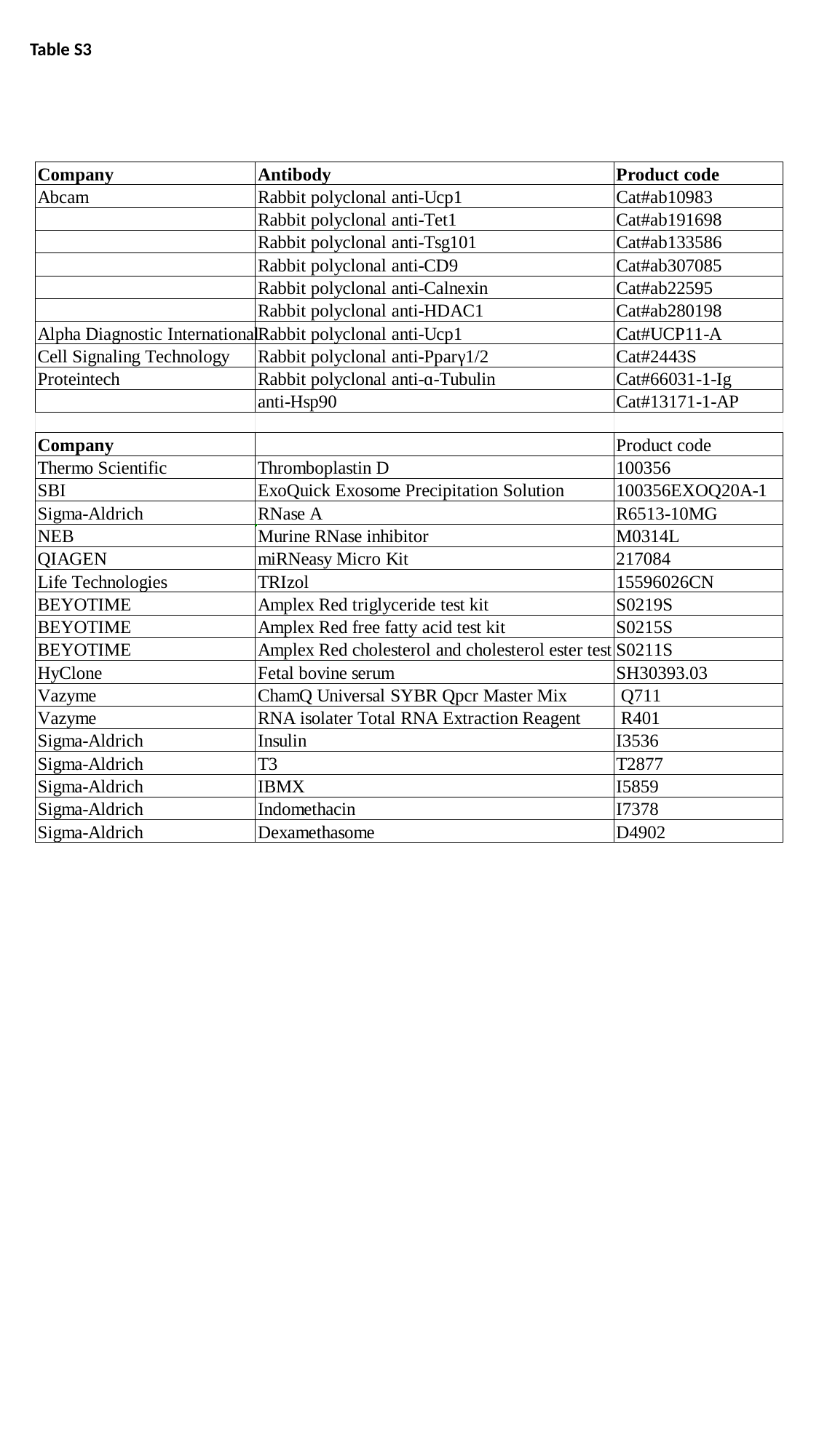

Table S3
