# Supplementary material for: Cold-induced hepatocyte-derived exosomes activate brown adipose thermogenesis via miR-293-5p-mediated transcriptional reprogramming
Source: Cell Death Discov. 2025 Aug 22;11:396. doi: 10.1038/s41420-025-02697-1 (PMC12373855; doi:10.1038/s41420-025-02697-1)
Supplement: Supplementary file 2 — wb data [file 41420_2025_2697_MOESM2_ESM.pptx]

## Slide 1
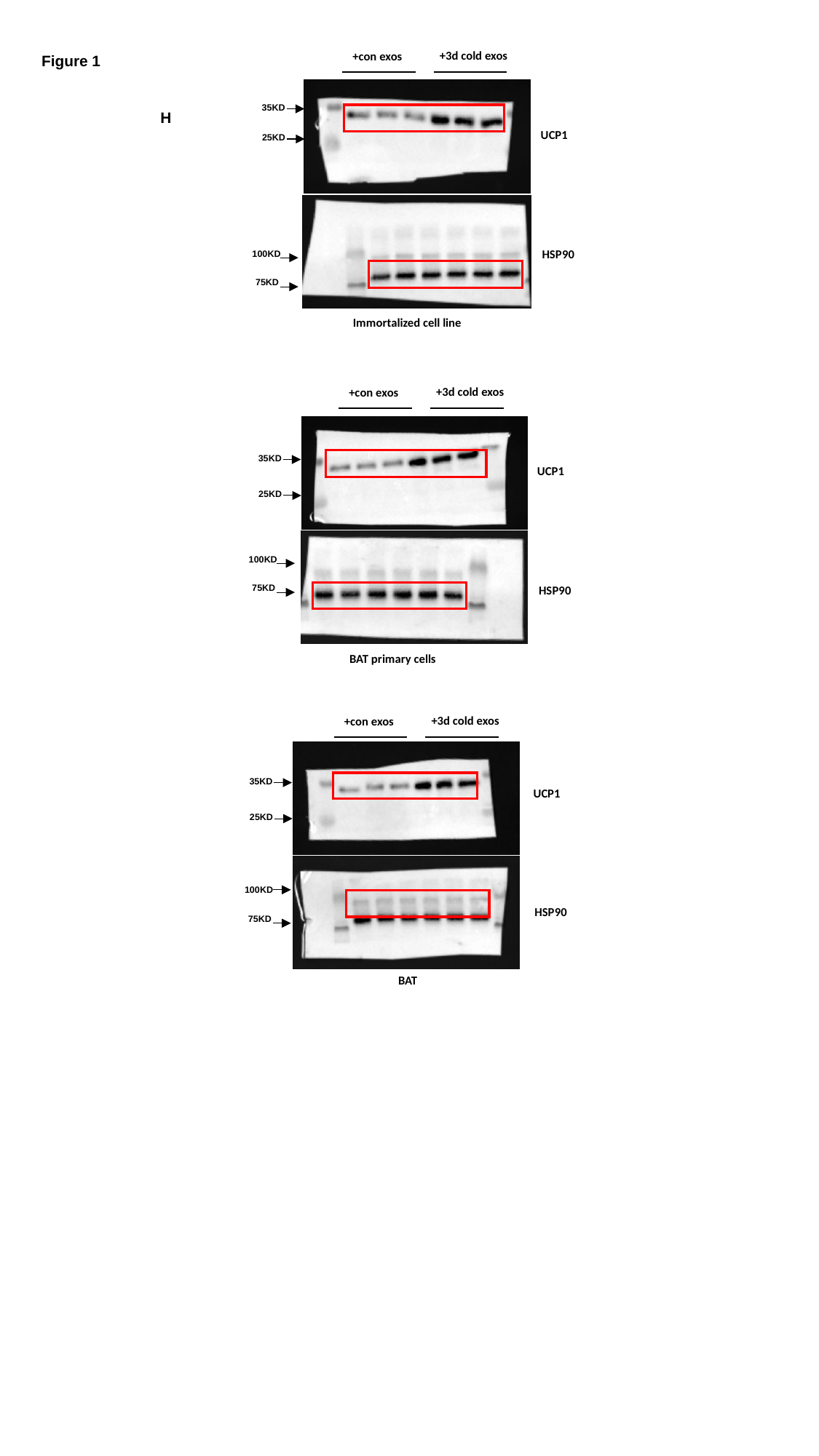

+3d cold exos
+con exos
35KD
25KD
UCP1
HSP90
100KD
75KD
Immortalized cell line
Figure 1
H
+3d cold exos
+con exos
35KD
UCP1
25KD
100KD
75KD
HSP90
BAT primary cells
+3d cold exos
+con exos
35KD
UCP1
25KD
100KD
75KD
HSP90
BAT

## Slide 2
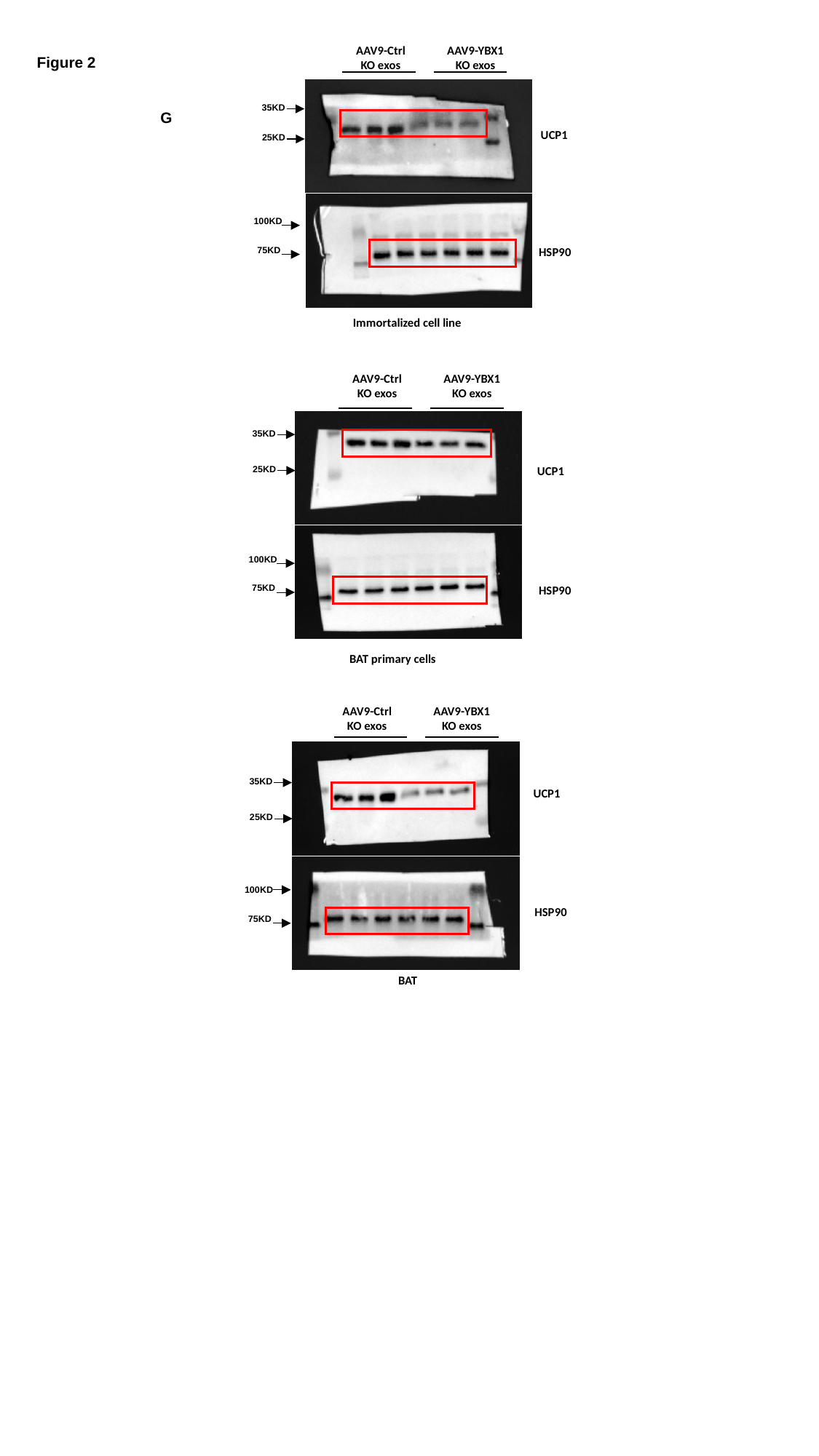

AAV9-Ctrl KO exos
AAV9-YBX1 KO exos
35KD
25KD
UCP1
100KD
75KD
HSP90
Immortalized cell line
Figure 2
G
AAV9-YBX1 KO exos
AAV9-Ctrl KO exos
35KD
25KD
UCP1
100KD
75KD
HSP90
BAT primary cells
AAV9-YBX1 KO exos
AAV9-Ctrl KO exos
35KD
UCP1
25KD
100KD
75KD
HSP90
BAT

## Slide 3
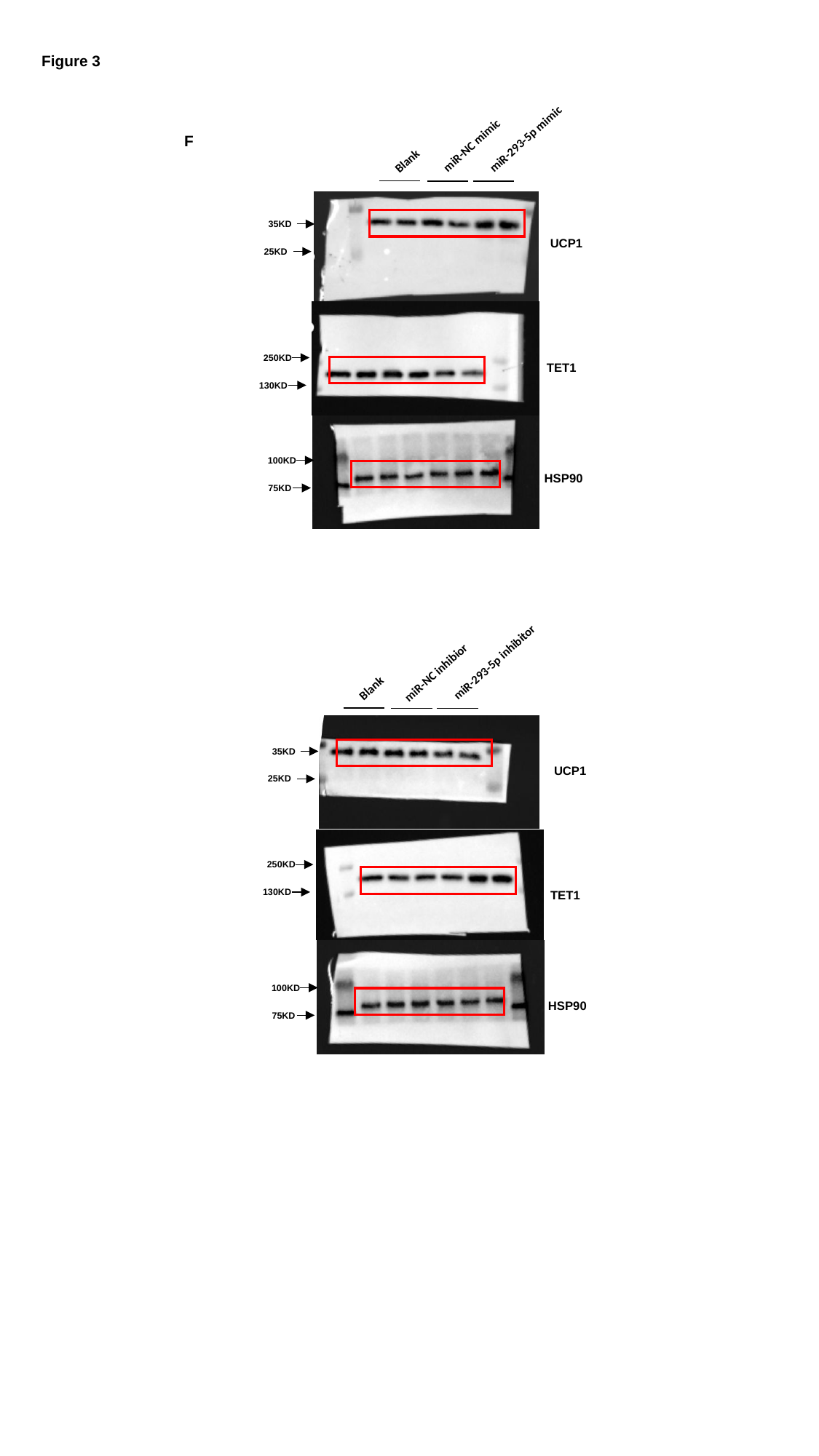

Figure 3
miR-293-5p mimic
miR-NC mimic
Blank
F
35KD
25KD
UCP1
250KD
130KD
TET1
100KD
75KD
HSP90
miR-293-5p inhibitor
miR-NC inhibior
Blank
35KD
25KD
UCP1
250KD
130KD
TET1
100KD
75KD
HSP90

## Slide 4
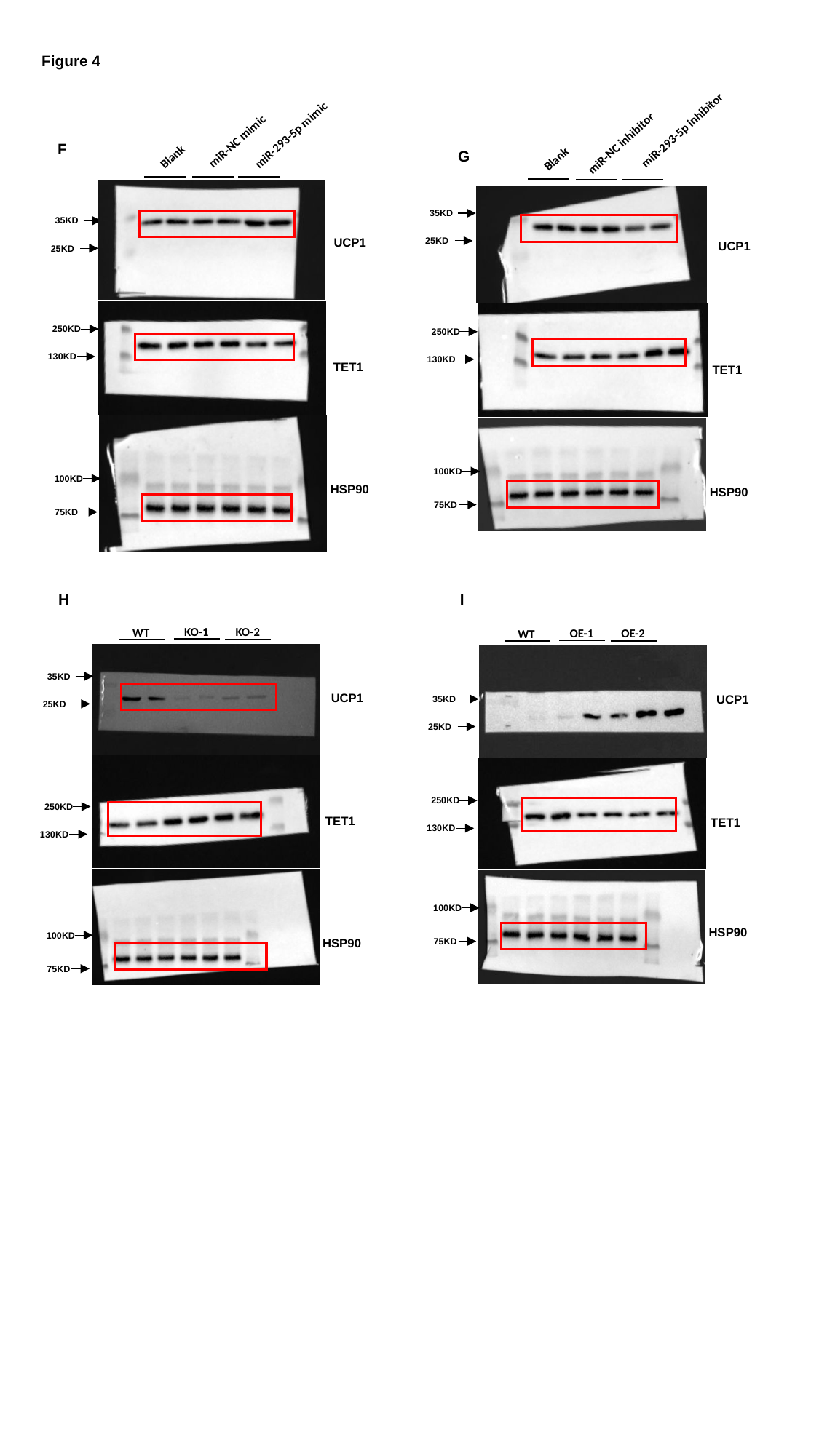

Figure 4
miR-293-5p inhibitor
miR-NC inhibitor
Blank
35KD
25KD
UCP1
250KD
130KD
TET1
100KD
HSP90
75KD
miR-293-5p mimic
miR-NC mimic
Blank
35KD
25KD
UCP1
250KD
130KD
TET1
100KD
HSP90
75KD
F
G
H
I
KO-2
KO-1
WT
35KD
25KD
UCP1
250KD
130KD
TET1
100KD
75KD
HSP90
OE-2
OE-1
WT
UCP1
35KD
25KD
250KD
130KD
TET1
100KD
75KD
HSP90

## Slide 5
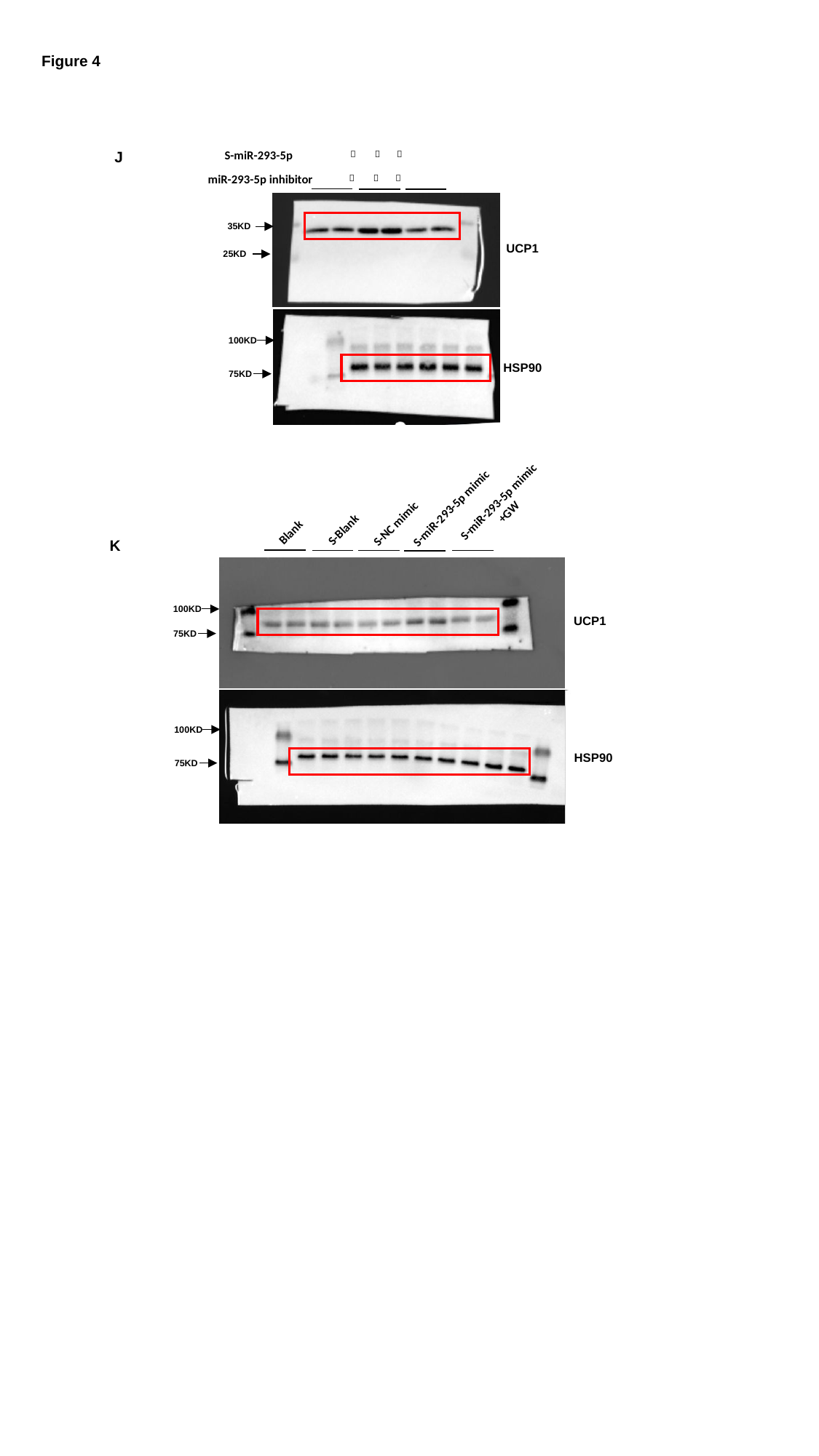

Figure 4
J
S-miR-293-5p
➖ ➕ ➕
➖ ➖ ➕
miR-293-5p inhibitor
35KD
25KD
UCP1
100KD
75KD
HSP90
S-miR-293-5p mimic +GW
S-miR-293-5p mimic
S-NC mimic
S-Blank
Blank
K
100KD
UCP1
75KD
100KD
75KD
HSP90

## Slide 6
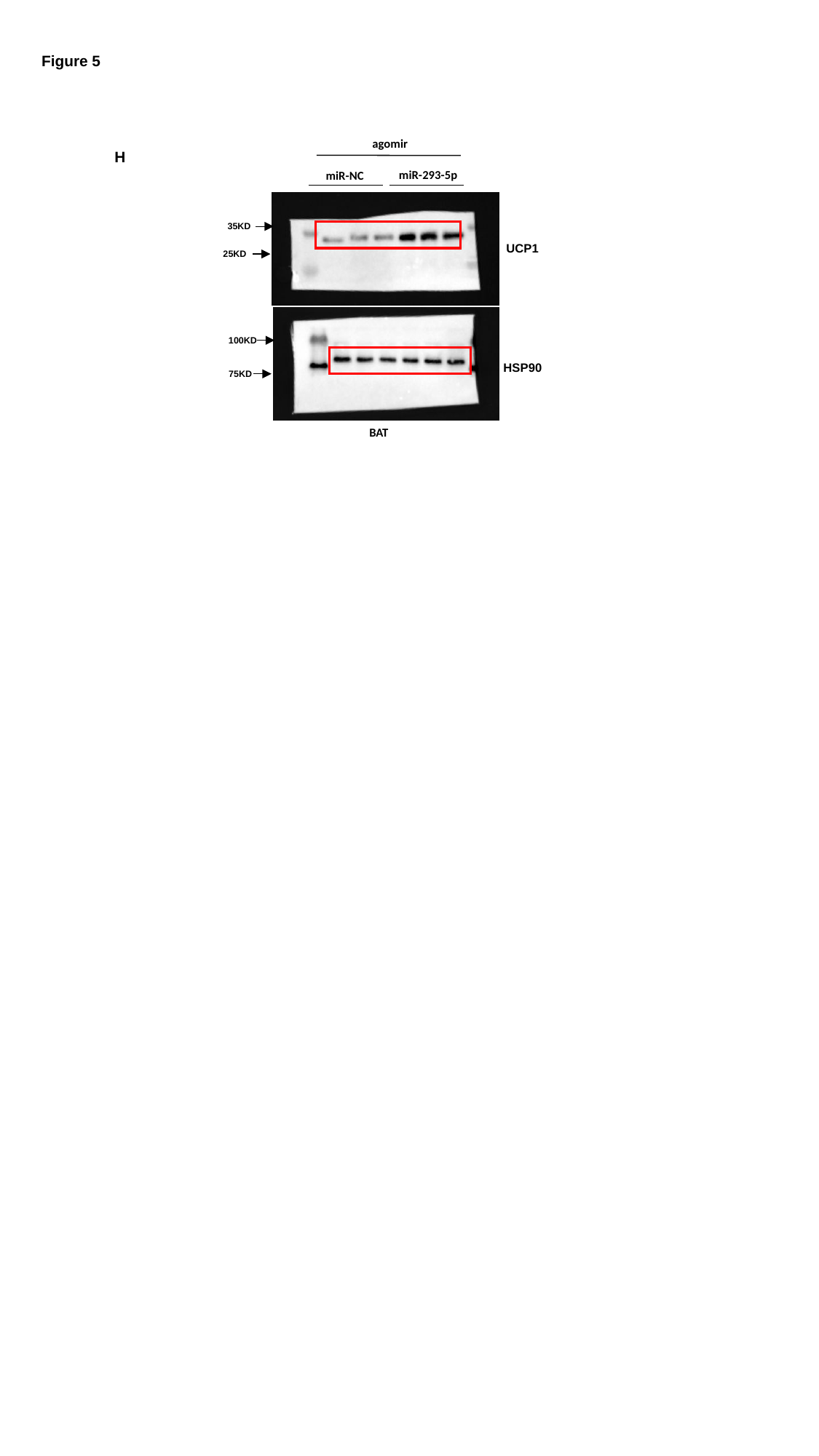

Figure 5
agomir
miR-293-5p
miR-NC
H
35KD
25KD
UCP1
100KD
75KD
HSP90
BAT

## Slide 7
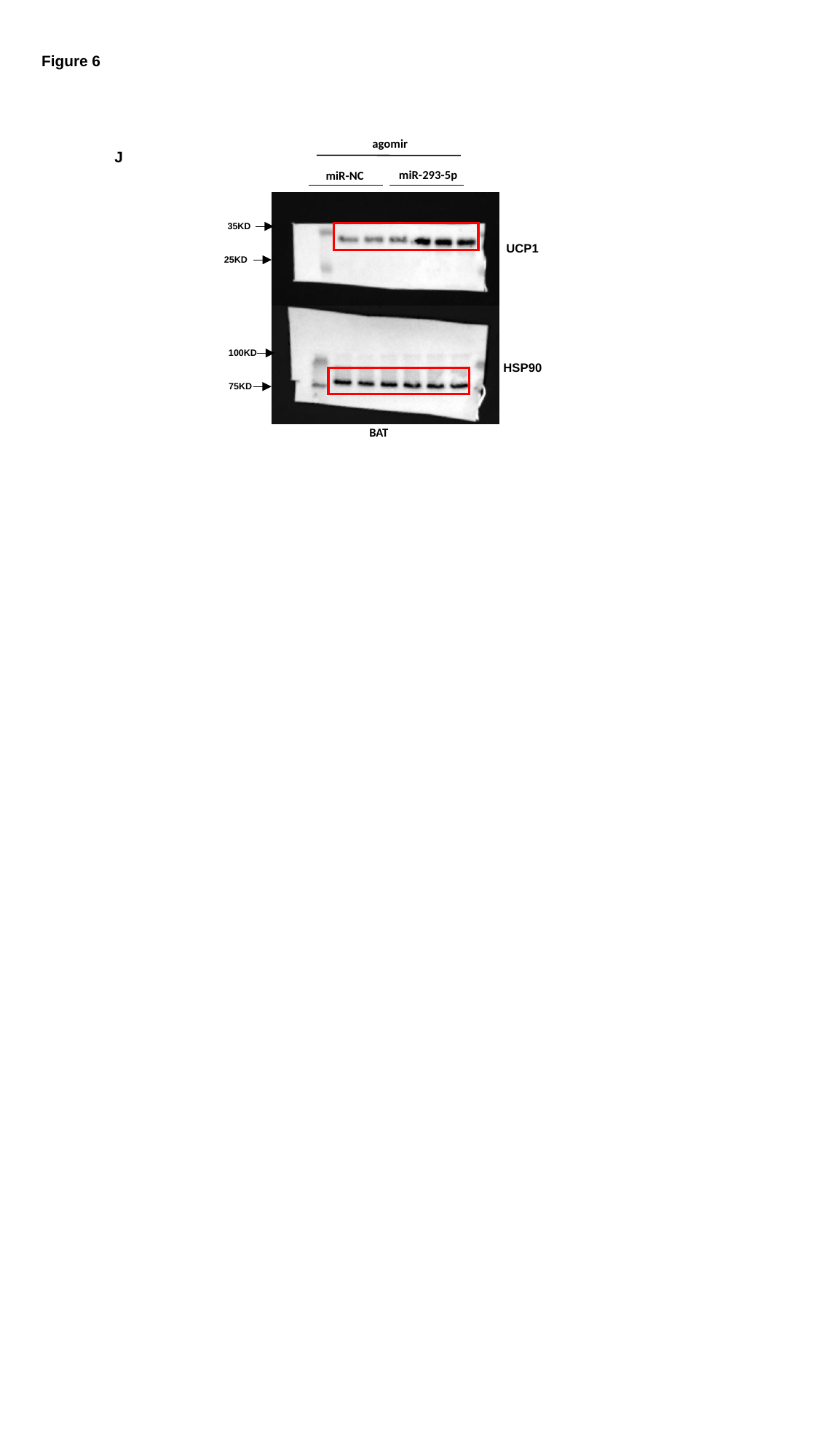

Figure 6
agomir
miR-293-5p
miR-NC
J
35KD
UCP1
25KD
100KD
75KD
HSP90
BAT

## Slide 8
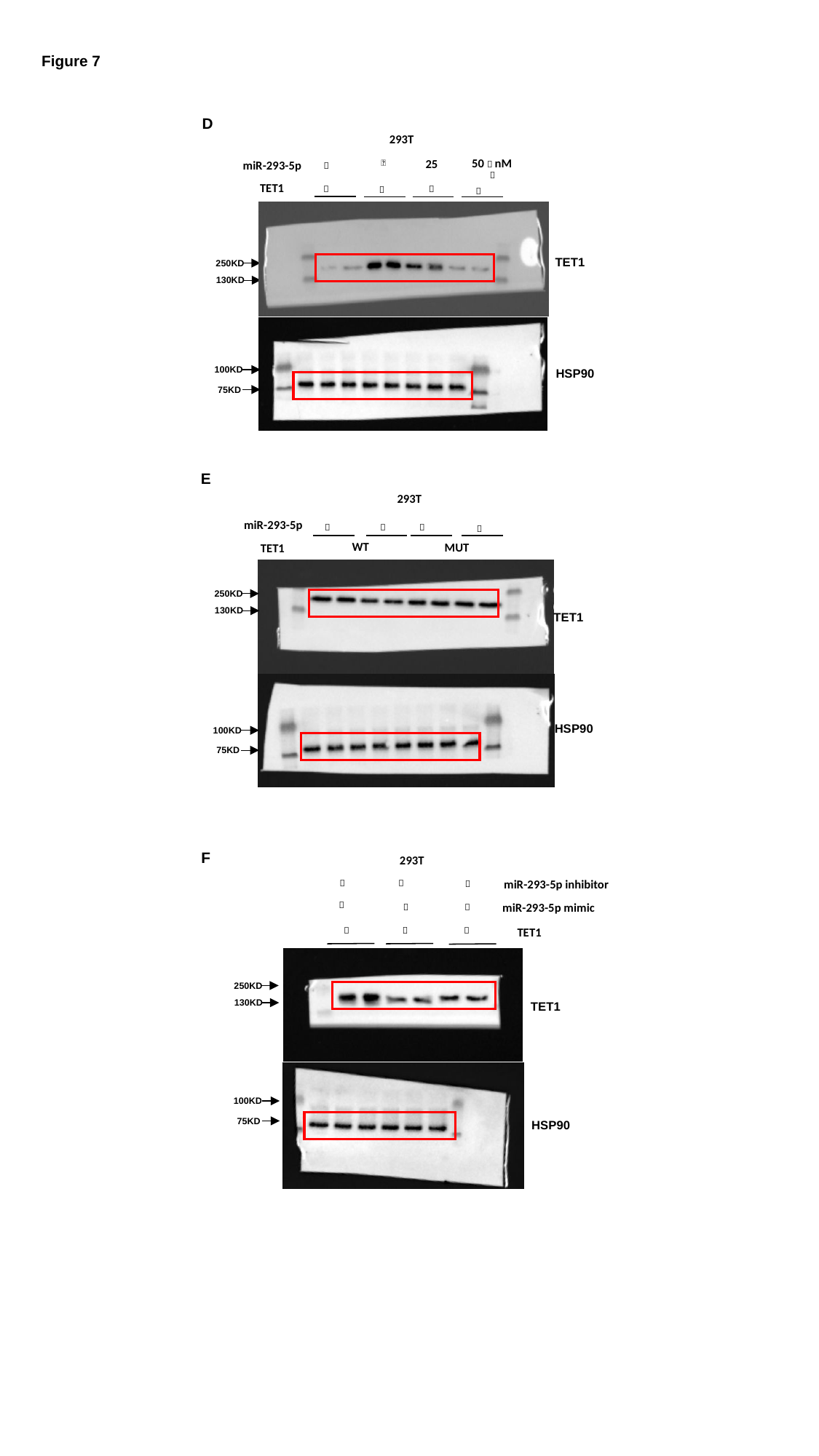

Figure 7
D
293T
50（nM）
25
➖
➖
miR-293-5p
TET1
➕
➕
➕
➖
TET1
250KD
130KD
HSP90
100KD
75KD
E
293T
miR-293-5p
➕
➕
➖
➖
WT
MUT
TET1
250KD
130KD
TET1
HSP90
100KD
75KD
F
293T
➕
➕
➕
miR-293-5p inhibitor
miR-293-5p mimic
TET1
➖
➕
➖
➖
➕
➕
250KD
130KD
TET1
100KD
75KD
HSP90

## Slide 9
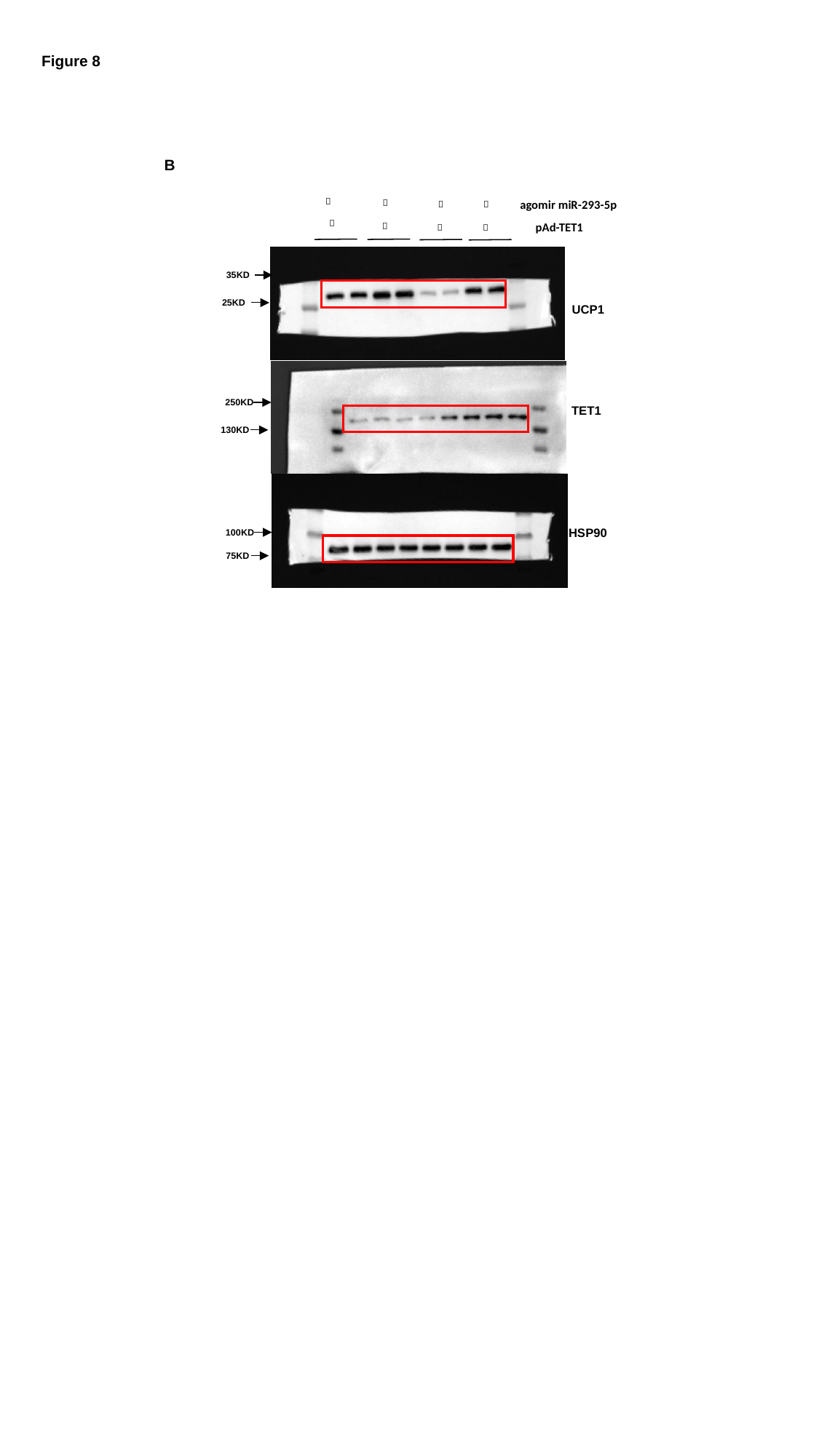

Figure 8
B
➕
➖
➖
➖
➕
➕
➖
➕
agomir miR-293-5p
pAd-TET1
35KD
25KD
UCP1
TET1
HSP90
250KD
130KD
100KD
75KD

## Slide 10
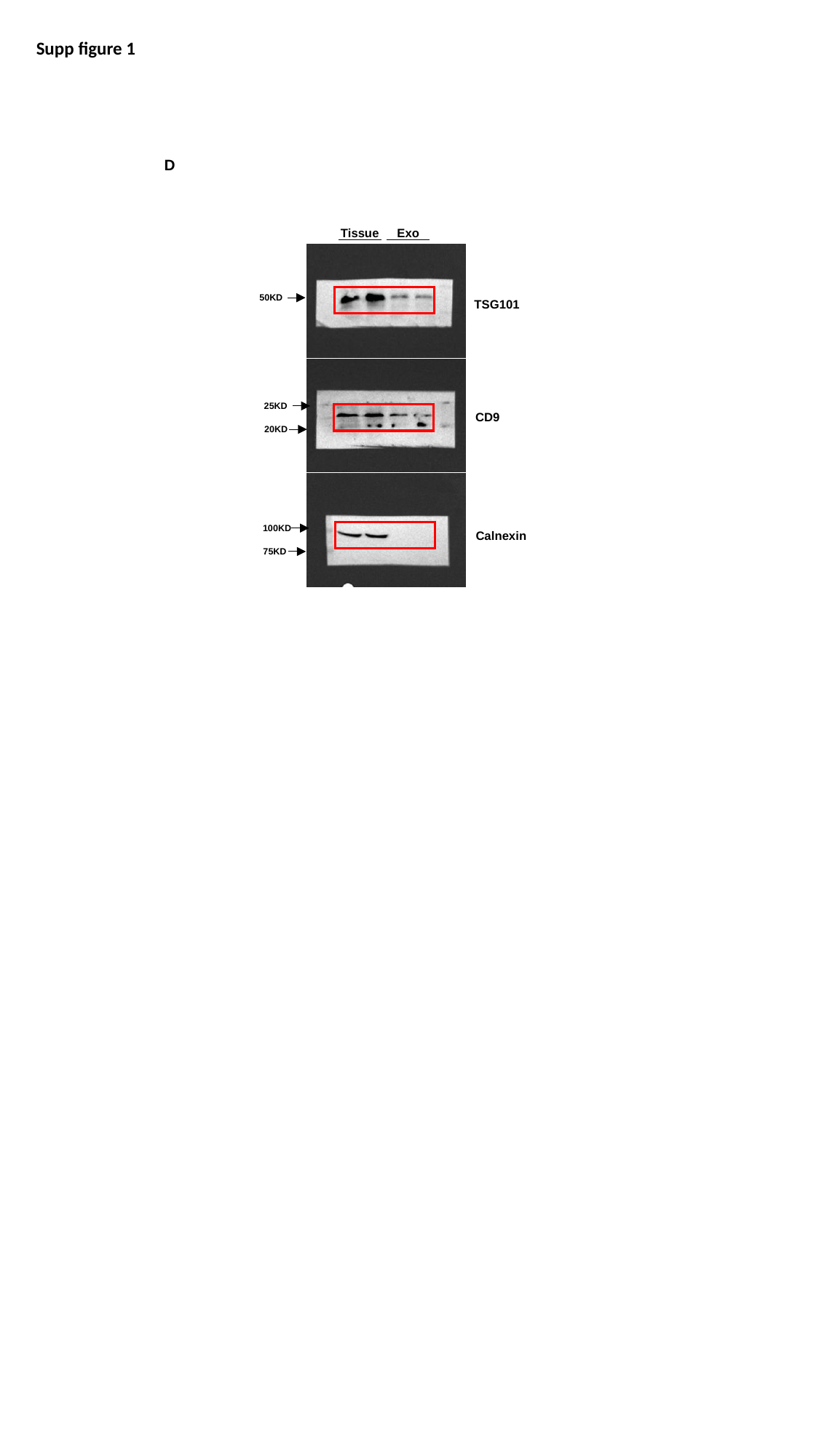

Supp figure 1
D
Tissue
Exo
TSG101
50KD
25KD
20KD
CD9
100KD
75KD
Calnexin

## Slide 11
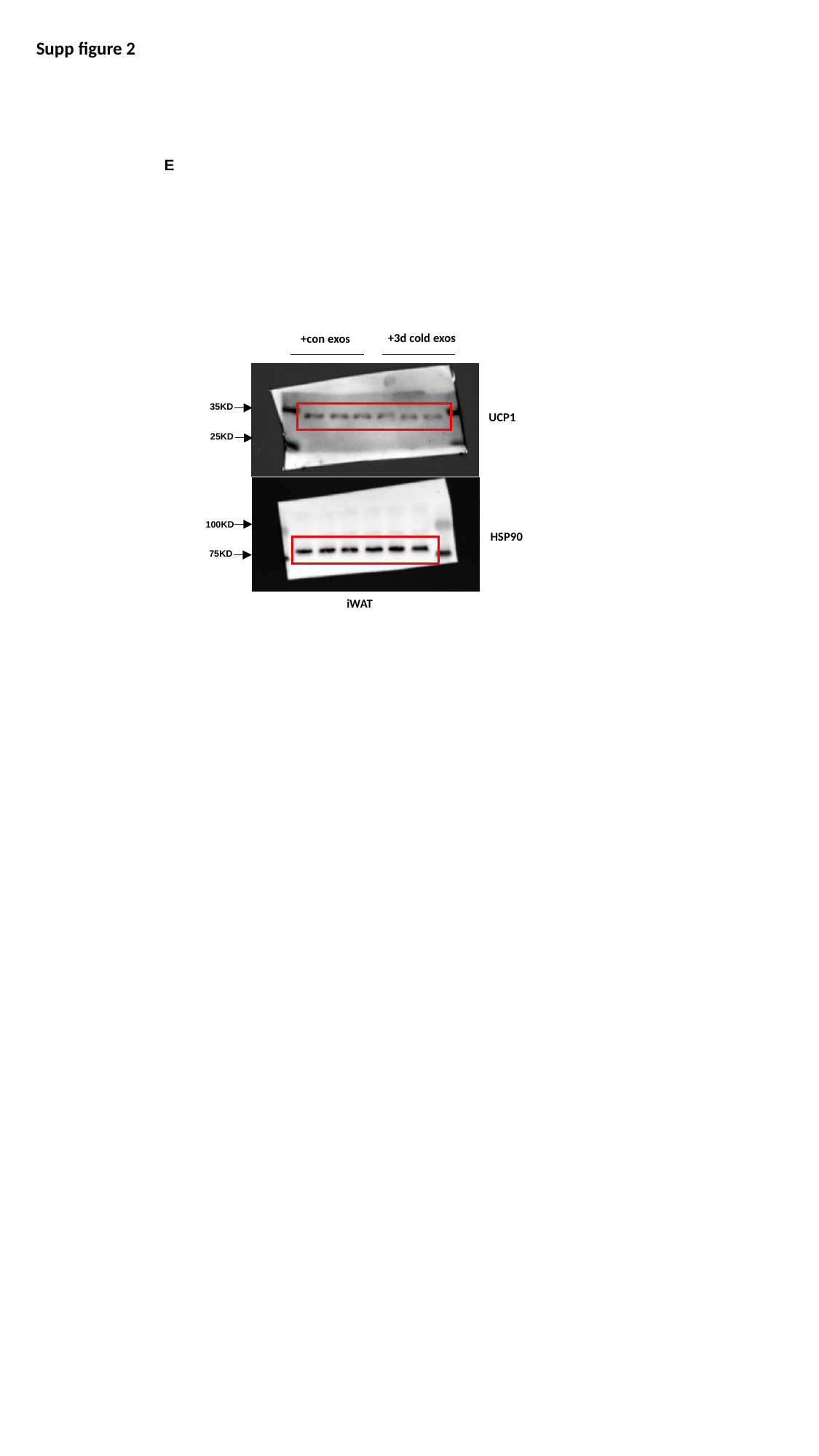

Supp figure 2
E
+3d cold exos
+con exos
35KD
25KD
UCP1
100KD
75KD
HSP90
iWAT

## Slide 12
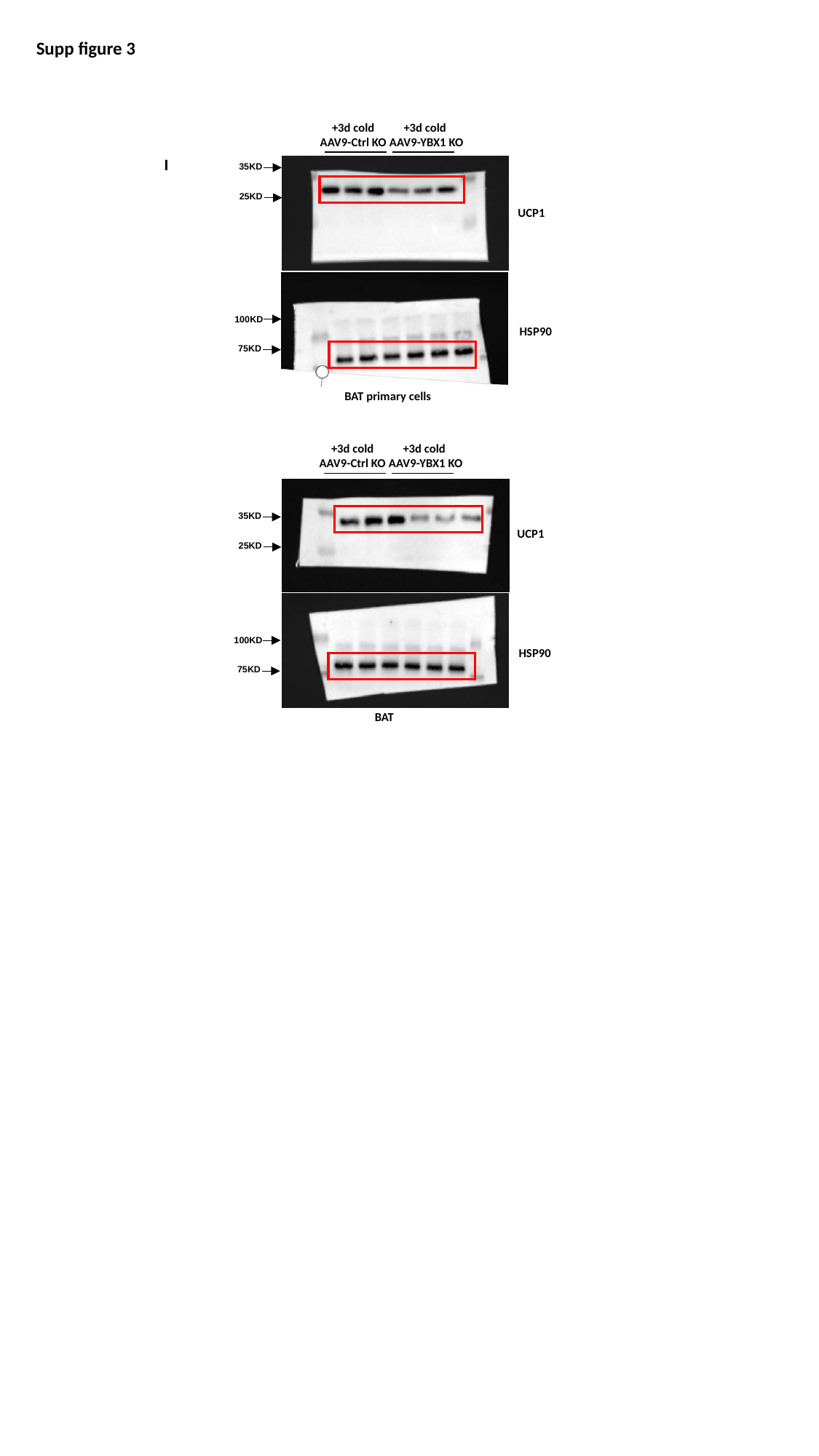

Supp figure 3
+3d cold
AAV9-YBX1 KO
+3d cold AAV9-Ctrl KO
35KD
25KD
UCP1
100KD
75KD
HSP90
BAT primary cells
I
+3d cold
AAV9-YBX1 KO
+3d cold AAV9-Ctrl KO
35KD
25KD
UCP1
100KD
75KD
HSP90
BAT

## Slide 13
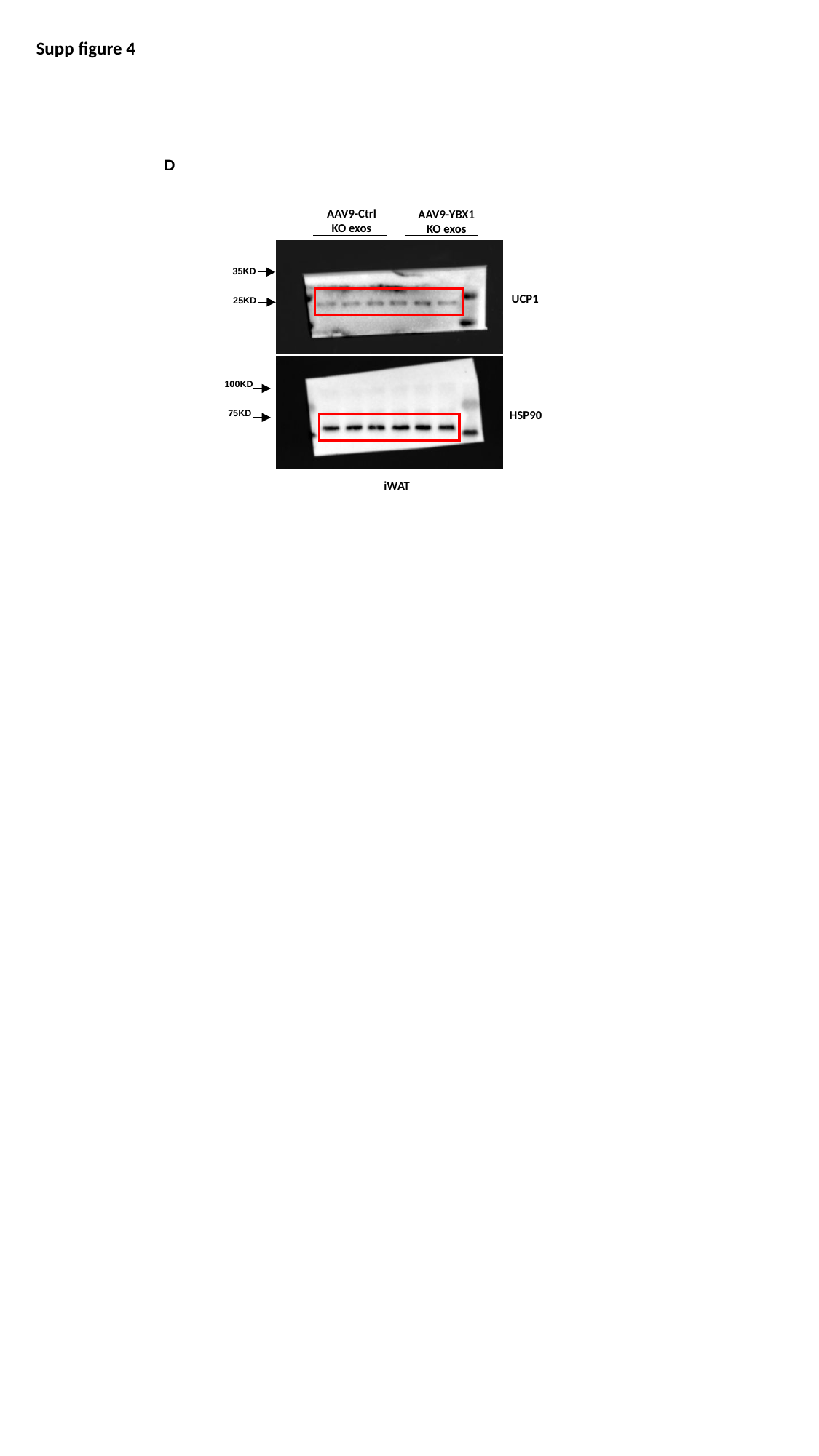

Supp figure 4
D
AAV9-Ctrl KO exos
AAV9-YBX1 KO exos
35KD
25KD
UCP1
100KD
75KD
HSP90
iWAT

## Slide 14
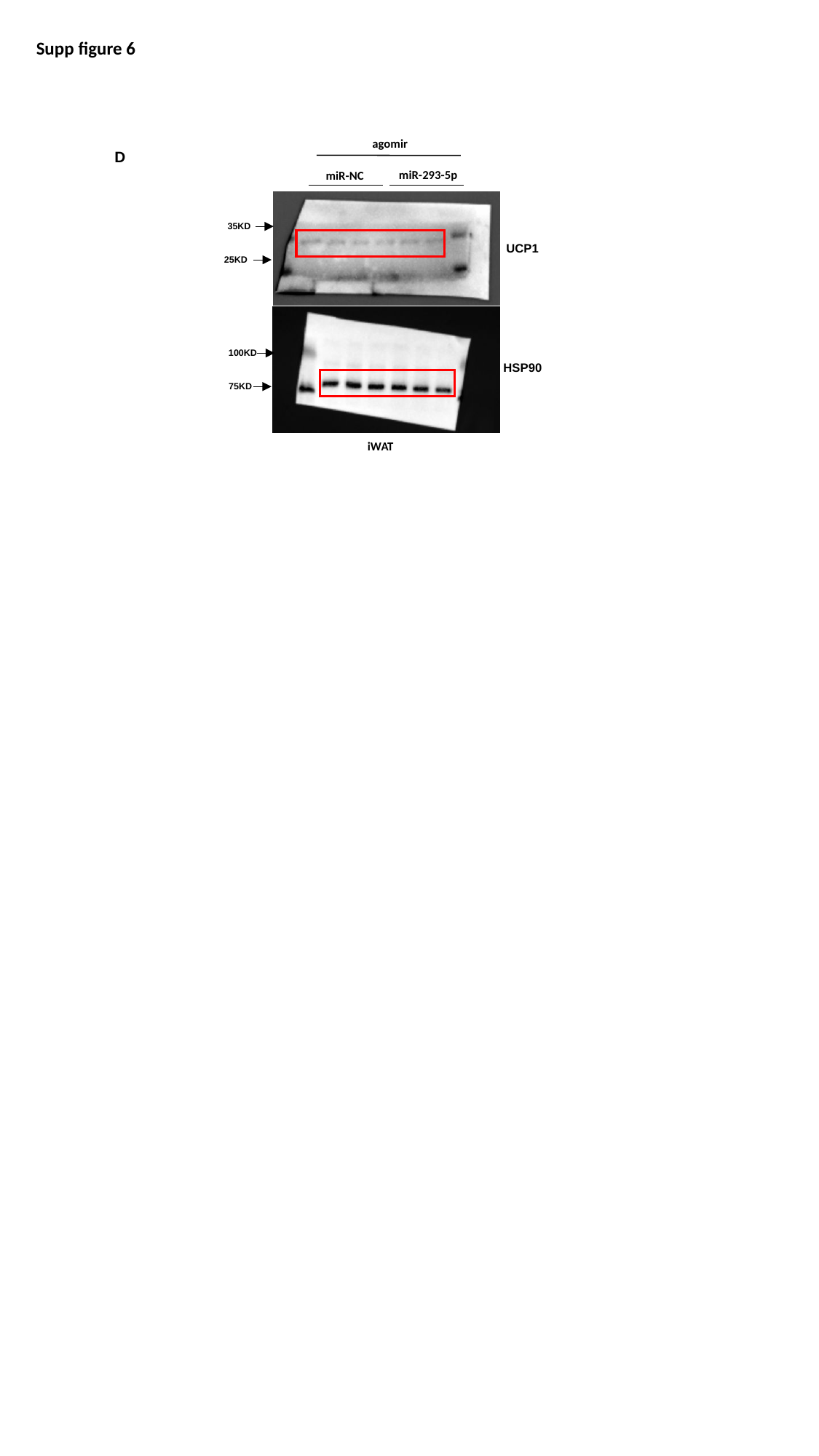

Supp figure 6
agomir
miR-293-5p
miR-NC
D
35KD
UCP1
25KD
100KD
75KD
HSP90
iWAT

## Slide 15
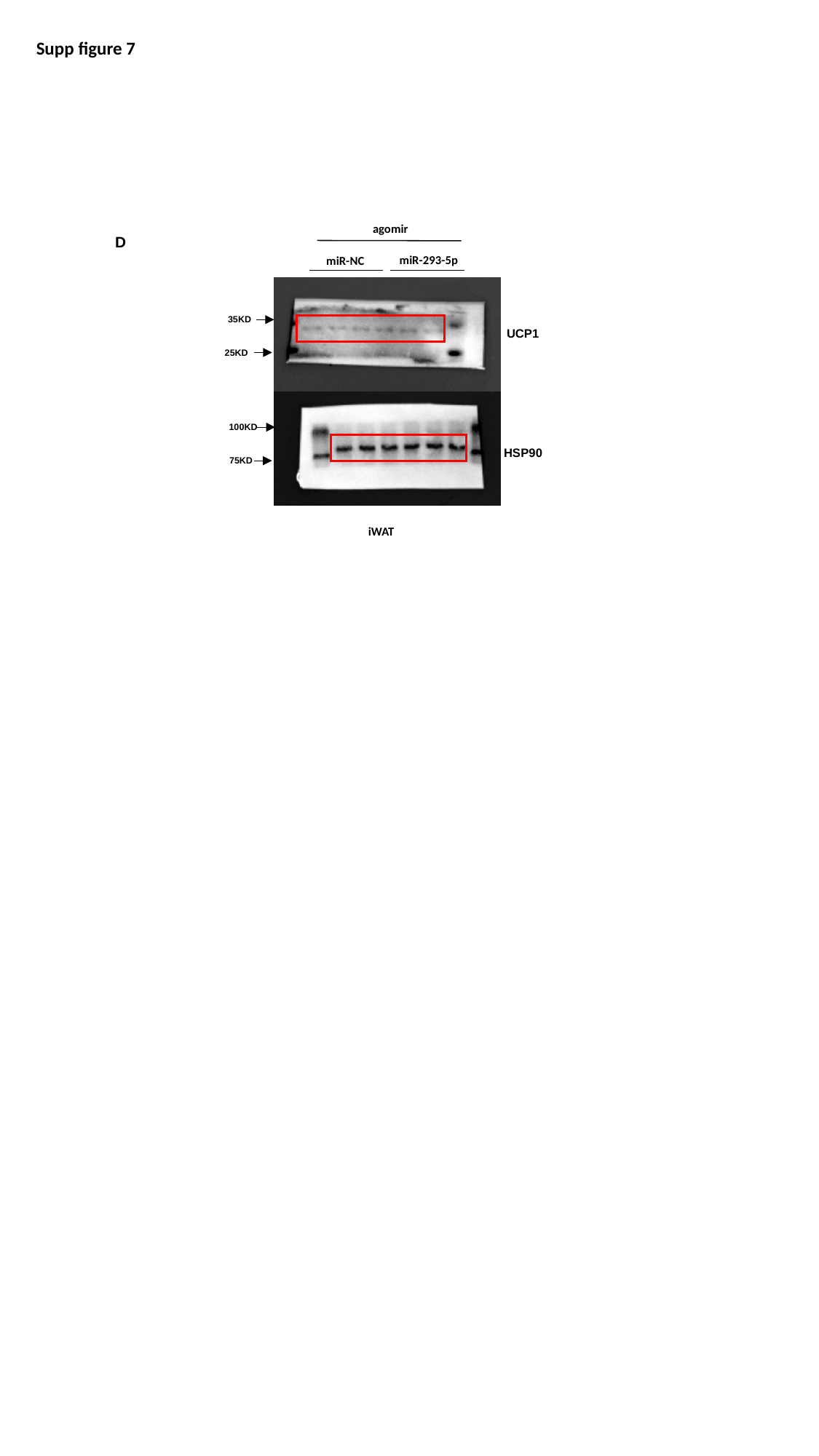

Supp figure 7
agomir
miR-293-5p
miR-NC
D
35KD
UCP1
25KD
100KD
75KD
HSP90
iWAT
