# Supplementary material for: Cold-induced hepatocyte-derived exosomes activate brown adipose thermogenesis via miR-293-5p-mediated transcriptional reprogramming
Source: Cell Death Discov. 2025 Aug 22;11:396. doi: 10.1038/s41420-025-02697-1 (PMC12373855; doi:10.1038/s41420-025-02697-1)
Supplement: Supplementary file 3 — Table S1 [file 41420_2025_2697_MOESM3_ESM.docx]

**Table S1 miRNA sequencing data**

| **BAT** | Cold1_count | Cold2_count | Cold3_count | Cold4_count | Control1_count | Control2_count | Control3_count | Control4_count |
| --- | --- | --- | --- | --- | --- | --- | --- | --- |
| mmu-miR-5106 | 230 | 218 | 360 | 446 | 66 | 176 | 126 | 342 |
| mmu-miR-293-5p | 1076 | 1680 | 1944 | 1734 | 242 | 395 | 397 | 345 |
| mmu-miR-3960 | 236 | 264 | 9149 | 7010 | 321 | 342 | 277 | 235 |
| mmu-miR-6374 | 20 | 16 | 28 | 32 | 5 | 17 | 19 | 16 |
| mmu-let-7f-5p | 405 | 316 | 579 | 487 | 1309 | 3094 | 1414 | 2456 |
| mmu-let-7a-5p | 662 | 462 | 1111 | 801 | 2540 | 5818 | 2616 | 2314 |
| mmu-let-7e-5p | 63 | 61 | 138 | 106 | 283 | 639 | 379 | 368 |
| mmu-miR-2137 | 194 | 229 | 456 | 489 | 1164 | 1155 | 1851 | 1765 |
| mmu-let-7d-5p | 161 | 133 | 225 | 142 | 535 | 895 | 638 | 765 |
| mmu-let-7c-5p | 2266 | 2457 | 3506 | 3085 | 7452 | 14675 | 10427 | 23143 |
| mmu-let-7g-5p | 180 | 144 | 240 | 190 | 534 | 928 | 556 | 657 |
| mmu-miR-98-5p | 10 | 7 | 24 | 20 | 47 | 74 | 62 | 73 |

| **BLOOD** | Cold1_count | Cold2_count | Cold3_count | Cold4_count | Control1_count | Control2_count | Control3_count | Control4_count |
| --- | --- | --- | --- | --- | --- | --- | --- | --- |
| mmu-miR-5126 | 234 | 234 | 117 | 94 | 817 | 594 | 632 | 359 |
| mmu-miR-6938-3p | 28 | 33 | 40 | 46 | 3 | 2 | 1 | 0 |
| mmu-miR-2137 | 120 | 136 | 124 | 120 | 523 | 476 | 294 | 235 |
| mmu-miR-3473b | 1680 | 1187 | 484 | 405 | 3024 | 2261 | 3511 | 1977 |
| mmu-miR-3473e | 1661 | 1168 | 476 | 398 | 2952 | 2225 | 3469 | 1934 |
| mmu-miR-5130 | 109 | 102 | 34 | 16 | 317 | 210 | 292 | 88 |
| mmu-miR-3106-5p | 68 | 65 | 96 | 107 | 32 | 16 | 10 | 8 |
| mmu-miR-5128 | 15 | 10 | 2 | 4 | 28 | 20 | 32 | 36 |
| mmu-miR-5106 | 642 | 652 | 104 | 166 | 83 | 69 | 80 | 52 |
| mmu-miR-16-5p | 1190 | 1185 | 1119 | 1333 | 98 | 79 | 46 | 26 |
| mmu-miR-12193-5p | 13 | 11 | 6 | 10 | 28 | 27 | 18 | 11 |
| mmu-miR-6990-5p | 137 | 100 | 111 | 128 | 58 | 32 | 48 | 36 |
| mmu-miR-29a-3p | 27 | 40 | 19 | 29 | 17 | 15 | 2 | 5 |
| mmu-miR-293-5p | 2211 | 2611 | 1623 | 1832 | 80 | 140 | 40 | 30 |

| **LIVER** | Cold1_count | Cold2_count | Cold3_count | Control1_count | Control2_count | Control3_count |
| --- | --- | --- | --- | --- | --- | --- |
| mmu-miR-122-5p | 42980 | 101065 | 55153 | 8746 | 13122 | 8889 |
| mmu-miR-16-5p | 102 | 247 | 179 | 24 | 16 | 53 |
| mmu-miR-293-5p | 12833 | 13232 | 15773 | 415 | 471 | 300 |
| mmu-miR-7665-5p | 35 | 63 | 33 | 164 | 151 | 124 |
| mmu-miR-674-5p | 14 | 22 | 12 | 7 | 0 | 4 |
| mmu-miR-7a-5p | 16 | 32 | 24 | 5 | 11 | 2 |
| mmu-miR-6769b-5p | 13 | 24 | 17 | 11 | 4 | 15 |
| mmu-miR-504-3p | 10 | 12 | 10 | 29 | 37 | 25 |
| mmu-miR-7081-5p | 31 | 72 | 46 | 15 | 31 | 52 |
| mmu-miR-7669-3p | 17 | 35 | 25 | 36 | 29 | 52 |
